# Sequential fate-switches in stem-like cells drive the tumorigenic trajectory from human neural stem cells to malignant glioma

**DOI:** 10.1038/s41422-020-00451-z

**Published:** 2021-01-04

**Authors:** Xiaofei Wang, Ran Zhou, Yanzhen Xiong, Lingling Zhou, Xiang Yan, Manli Wang, Fan Li, Chuanxing Xie, Yiming Zhang, Zongyao Huang, Chaoqiong Ding, Kaidou Shi, Weida Li, Yu Liu, Zhongwei Cao, Zhen-Ning Zhang, Shengtao Zhou, Chong Chen, Yan Zhang, Lu Chen, Yuan Wang

**Affiliations:** 1 Department of Neurology and Department of Neurosurgery, State Key Laboratory of Biotherapy and Cancer Center, West China Hospital, Sichuan University and National Collaborative Innovation Center, Chengdu, Sichuan 610041 China; 2 Key Laboratory of Birth Defects and Related Diseases of Women and Children of MOE, State Key Laboratory of Biotherapy, West China Second Hospital, Sichuan University, Chengdu, Sichuan 610041 China; 3 National Clinical Research Center for Geriatrics, State Key Laboratory of Biotherapy, West China Hospital, Sichuan University, Chengdu, Sichuan 610041 China; 4 Department of Hematology, State Key Laboratory of Biotherapy, West China Hospital, Sichuan University, Chengdu, Sichuan 610041 China; 5 Institute of Regenerative Medicine, Shanghai East Hospital, Tongji University, Shanghai, 200092 China

**Keywords:** CNS cancer, Cancer models, Cancer stem cells, Tumour heterogeneity

## Abstract

Glioblastoma (GBM) is an incurable and highly heterogeneous brain tumor, originating from human neural stem/progenitor cells (hNSCs/hNPCs) years ahead of diagnosis. Despite extensive efforts to characterize hNSCs and end-stage GBM at bulk and single-cell levels, the de novo gliomagenic path from hNSCs is largely unknown due to technical difficulties in early-stage sampling and preclinical modeling. Here, we established two highly penetrant hNSC-derived malignant glioma models, which resemble the histopathology and transcriptional heterogeneity of human GBM. Integrating time-series analyses of whole-exome sequencing, bulk and single-cell RNA-seq, we reconstructed gliomagenic trajectories, and identified a persistent NSC-like population at all stages of tumorigenesis. Through trajectory analyses and lineage tracing, we showed that tumor progression is primarily driven by multi-step transcriptional reprogramming and fate-switches in the NSC-like cells, which sequentially generate malignant heterogeneity and induce tumor phenotype transitions. We further uncovered stage-specific oncogenic cascades, and among the candidate genes we functionally validated C1QL1 as a new glioma-promoting factor. Importantly, the neurogenic-to-gliogenic switch in NSC-like cells marks an early stage characterized by a burst of oncogenic alterations, during which transient AP-1 inhibition is sufficient to inhibit gliomagenesis. Together, our results reveal previously undercharacterized molecular dynamics and fate choices driving de novo gliomagenesis from hNSCs, and provide a blueprint for potential early-stage treatment/diagnosis for GBM.

## Introduction

Glioblastoma (GBM, World Health Organization WHO Grade IV) is the most common and aggressive primary brain cancer with a median survival of 15 months.^1,2^ GBM is among the best molecularly characterized cancer types, leading to recognition of its extreme inter- and intra-tumor heterogeneity. Bulk GBMs can be classified into at least three molecular subtypes, namely Proneural, Classical and Mesenchymal.^3,4^ Recent single-cell analyses further determined that GBMs exist in diverse cellular states, and contain heterogeneous stem-like subpopulations.^5–8^ Despite these efforts, the prognosis of GBM remains unimproved in the past decade.

One reason for the poor prognosis of GBM may be delayed diagnosis and treatment. Over 90% of GBMs are primary GBMs, which are full-blown tumors at diagnosis without clinical proof of pre-existing lower-grade lesions. However, genetic evidence postulates that they may arise from an undetectable cell of origin several years before initial diagnosis.^9^ The most likely cell(s) of origin for GBM are neural stem/progenitor cells (NSCs/NPCs) in the subventricular zone (SVZ) and oligodendrocyte precursor cells (OPCs), as demonstrated by studies on genetically engineered mouse models (GEMMs).^10–14^ In support of the NSC-origin hypothesis, a recent study showed that non-tumor-associated human SVZ contains low-level driver mutations shared with matched GBM in distant brain regions, indicating an NSC-to-GBM evolution.^15^ However, the de novo tumorigenic path from human NSCs (hNSCs) towards heterogeneous GBM cells during the long period of tumor latency remains largely unknown, and it is yet to be determined whether there is a window of opportunity for early-stage diagnosis and preventative treatment of GBM.

Since de novo gliomagenesis is a process that precedes the development of full-blown tumors, it cannot be investigated in well-established pre-clinical models generated from end-stage tumors, such as tumor cell lines, tumorspheres, or patient-derived xenograft models including glioma stem cell (GSC) xenograft models. It is also technically challenging to collect early-stage patient samples. Recent advances in human stem cell biology and genome-editing techniques provide new tools to address this question,^16^ allowing for direct glioma modeling from human stem cells by introducing defined initiating mutations.^17,18^ A recent study established glioma models from human induced pluripotent stem cells, performed longitudinal analysis on primary tumorspheres, secondary tumors, and secondary tumorspheres, and revealed how tumor cells evolve between in vivo and in vitro passages.^17^ However, the de novo gliomagenic trajectory from hNSCs is yet to be determined in highly penetrant hNSC-derived glioma models.

In this study, we established two orthotopic malignant glioma models from genome-edited hNSCs with 94%–100% penetrance, which resemble histopathological features and transcriptional heterogeneity of human GBM at bulk and single-cell levels. Integrating multi-omic time-series analyses of deep whole exome sequencing (WES), bulk and single-cell RNA-seq (scRNA-seq), we reconstructed de novo tumorigenic trajectories from hNSCs. We show that tumor progression is primarily driven by multi-step transcriptional reprogramming in a persistent NSC-like population, while additional genetic alterations do not appear to play a dominant role. NSC-like cells exhibit stage-specific fate-switches and transcriptional alterations to generate malignant heterogeneity, leading to tumor phenotype transitions. Among top upregulated oncogenic candidates, we functionally validated C1QL1 as a new glioma-promoting factor. We further determined that the neurogenic-to-gliogenic switch in NSC-like cells marks an important early stage with a burst of oncogenic alterations including the upregulation of AP-1. Transient AP-1 inhibition at this stage was sufficient to impede gliomagenesis in vivo, providing a proof of concept for potential early-stage interventions against the gliomagenic trajectory.

## Results

### Genome-edited hNSCs with GBM-relevant tumor suppressor mutations generate malignant gliomas with high penetrance

**Fig. 1:**
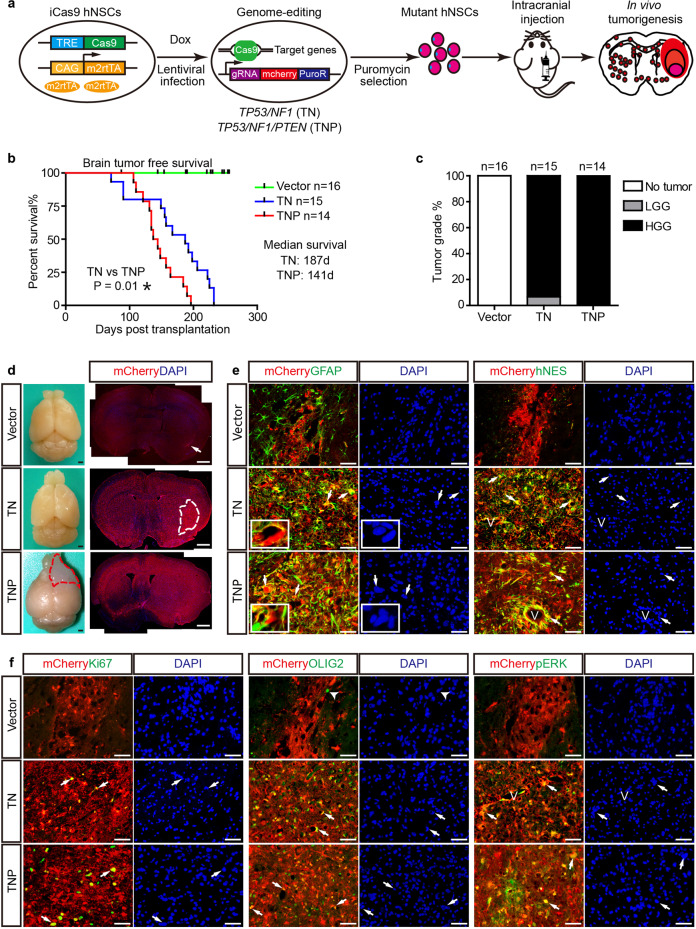
TN and TNP hNSCs generate HGGs in vivo with high penetrance. **a** Workflow to generate HGGs from iCas9 hNSCs. **b** The brain-tumor-free survival curves of Vector, TN, and TNP mice. n, the number of animals. *P* value, Log-rank (Mantel-cox) test. **c** The percentage of mice diagnosed with no tumor, LGG, and HGG in control and mutant groups. **d** Left: whole-mount view of end-stage Vector, TN, and TNP brains. Red dashed lines, an overt tumor in the TNP brain. Right: low magnification view of the progeny of transplanted cells marked by mCherry/DAPI in coronal brain sections at the end stage. Arrow, mCherry^+^ cells in the Vector brain. White dashed lines, the central focal tumor area with high cellularity in the TN brain. **e**, **f** Immunofluorescence (IF) co-labeling of mCherry with GFAP, hNES, Ki67, OLIG2, and pERK on serial brain sections from end-stage Vector, TN, and TNP mice. DAPI, nuclei. Insets, mCherry^+^GFAP^+^ tumor cells surrounding the neuronal nuclei. Arrows, co-labeled cells. Arrowheads, OLIG2^+^mCherry^–^ cells. V, blood vessel. Scale bars, 1 mm (**d**); 50 μm (**e**, **f**).

To directly target GBM-relevant tumor suppressor mutations into hNSCs, we generated iCas9 hNSCs from Hues8-iCas9 human pluripotent stem cells (hPSCs), which contain doxycycline (dox)-inducible Cas9 transgene allowing for highly efficient one-step editing of multiple genes.^19^ We designed two combinations of gRNAs targeting *TP53*, *NF1*, *or PTEN* (Fig. 1a; Supplementary information, Fig. S1a, and Materials and Methods), which are among the top five mutated genes in GBM.^20^ gRNAs targeting *TP53*/*NF1*/*PTEN* (TNP) or *TP53*/*NF1* (TN) were introduced into iCas9 hNSCs in a lentiviral vector V2TC, which carries mCherry as a lineage tracing reporter. hNSCs transfected with an empty vector were used as controls (hereafter, Vector).

To avoid extended in vitro culture of genome-edited hNSCs which might accumulate undesired genetic/epigenetic alterations, we chose not to perform standard single-cell colony selection but relied on puromycin selection for viral transfected cells (Fig. 1a, Materials and Methods). Nevertheless, the mutation frequency of *TP53*, *NF1*, and *PTEN* in resultant hNSCs was over 93%, evidenced by western blot or endonuclease T7 assays (Supplementary information, Fig. S1b, c). Of note, while the protein levels of TP53 in TN and TNP hNSCs were only slightly reduced, the insertion/deletion (Indel) ratio at genome-edited loci was over 97%, suggesting that our targeting strategy may result in the expression of mutant TP53 proteins. In support of the high mutation frequency, TN and TNP hNSCs were consistently more clonogenic than Vector controls in colony formation assays (Supplementary information, Fig. S1d). The neural stem/progenitor cell identities of Vector, TN, and TNP hNSCs were confirmed by their robust expression of SOX2, PAX6, and human-specific NESTIN (hNES) (Supplementary information, Fig. S1e). Thus, we efficiently generated mutant hNSCs harboring multiple GBM-relevant tumor suppressor mutations.

To test whether genome-edited hNSCs can generate brain tumors in vivo, we orthotopically transplanted Vector, TN, and TNP hNSCs into the brains of immunodeficient NOD/SCID mice. None of the Vector mice developed brain tumors (0/16). In contrast, TN and TNP mice exhibited neurological symptoms requiring sacrifice between 2.5 and 8 months post transplantation. 100% of TN (15/15) and TNP (14/14) mice developed brain tumors, with statistically different median brain-tumor-free survival of 187 and 141 days, respectively (Fig. 1b, c). TNP but not TN tumors were consistently visible at the macroscopic level (Fig. 1d). Based on WHO criteria, 94% of TN (14/15) and 100% of TNP tumors (14/14) exhibited classical features of high-grade gliomas (HGGs) including anaplastic astrocytomas and GBMs, such as diffuse infiltrative growth, high degrees of nuclear atypia, mitosis, microvascular proliferation, and secondary structures of Scherer (Fig. 1c–f; Supplementary information, Fig. S1f). These tumors expressed high levels of glioma markers GFAP, hNES, Ki67, and pERK (Fig. 1e,f). OLIG2, a transcription factor (TF) and master regulator of glial fate and gliomagenesis,^21–23^ was also highly expressed (Fig. 1f). In contrast, the progeny of transplanted Vector hNSCs did not expand in the brains of age-matched mice, and barely expressed hNES, Ki67, OLIG2, or pERK (Fig. 1e, f). The majority of the cells expressed astrocyte marker GFAP, indicating astrocytic differentiation (Fig. 1e).

**Fig. 2:**
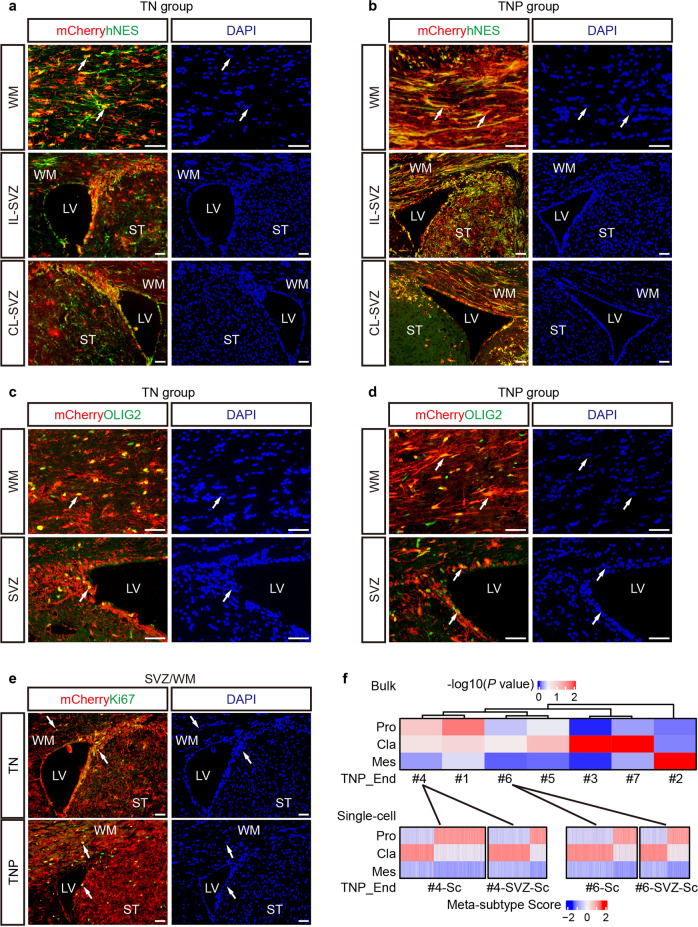
TN and TNP tumors histologically and transcriptionally resemble human GBM. **a**, **b** IF for mCherry/hNES in the WM and SVZ ipsilateral (IL) and contralateral (CL) to the transplantation sites in end-stage TN (**a**) and TNP (**b**) brains. **c**–**e** IF for mCherry/OLIG2 (**c, d**) and mCherry/Ki67 (**e**) in the WM and SVZ of end-stage TN and TNP brains. Arrows, co-labeled cells. LV, lateral ventricle. ST, striatum. WM, white matter. Scale bars, 50 μm. **f** Top: Heatmap of the *P* values (–log_10_) of GBM subtypes for bulk TNP samples at the end stage based on ssGSEA analysis. Bottom: Heatmap of the meta-subtype score for each single cell in TNP End and EndSVZ samples. Lines link samples from the same mouse.

Another hallmark feature of GBM is its high invasiveness. TN and TNP HGGs all exhibited extensive neoplastic infiltration, invading brain regions both ipsilateral and contralateral to the transplantation site (Fig. 1d). While TN tumors still retained a central focal tumor area around the transplantation site, TNP tumors diffusely infiltrated the entire anterior forebrain with minimal or no central focal area, reminiscent of some extreme cases of human GBM (Fig. 1d). In both TN and TNP mice, mCherry^+^hNES^+^ cells with elongated nuclei were readily identified around the blood vessels and along the white matter tract, the most common invasion routes of GBM^24^ (Figs. 1e, 2a, b). Importantly, TN and TNP tumors, but not Vector hNSCs, frequently invaded bilateral SVZs of the host mice, a phenomenon associated with increased recurrence and decreased survival in patients with GBM (Fig. 2a, b; Supplementary information, Fig. S2a, b).^25,26^ A significant portion of invading cells in the white matter and the SVZ expressed OLIG2 and Ki67 (Fig. 2c–e). In addition, transdifferentiated mCherry^+^αSMA^+^ pericytes and mCherry^+^hCD31^+^ endothelial cells were observed at the invasive fronts (Supplementary information, Fig. S2c, d). This is consistent with previous publications that human GBM cells can generate pericytes and/or endothelial cells to promote neovascularization.^27,28^ Together, these results demonstrate that genome-edited hNSCs with GBM-relevant tumor suppressor mutations generate malignant gliomas with high penetrance, which resemble pathological features of human GBM.

### TN and TNP tumors resemble inter- and intra-tumor heterogeneity of GBM

To investigate whether TN and TNP tumors transcriptionally mimic human GBM, we performed bulk RNA-seq in 6 TN and 11 TNP HGGs, and compared them to the RNA-seq from TCGA using GEPIA2.^29^ Both TN and TNP tumors were classified as GBM/Low-grade glioma (LGG) among 20 cancer types in the TCGA database (Supplementary information, Fig. S2e). To further determine their molecular subtypes, we used a published single sample gene set enrichment analysis (ssGSEA)-based strategy.^4^ Both TN and TNP tumors exhibited significant inter-tumor heterogeneity, representing all three TCGA subtypes (Fig. 2f; Supplementary information, Fig. S2f). To determine whether these tumors also exhibit intra-tumor heterogeneity, we performed paired scRNA-seq on two TNP tumors (#4 and #6) and their tumor-associated SVZs. Consistent with previous reports,^4,8^ individual TNP tumor cells expressed distinct subtype signatures, and the dominant subtype of single cells was the same as the bulk subtype (Fig. 2f). Notably, the majority of single cells from #4 SVZ were Classical, which is different from the paired Proneural non-SVZ tumor at bulk or single-cell level, highlighting the regional differences within the same tumor (Fig. 2f). In sum, these data indicate that genome-edited hNSCs with defined initiating mutations can give rise to malignant gliomas with inter- and intra-tumor heterogeneity, transcriptionally resembling human GBM at bulk and single-cell levels.

### A stage of “oncogenic burst” distinguishes de novo tumorigenesis from normal differentiation

**Fig. 3:**
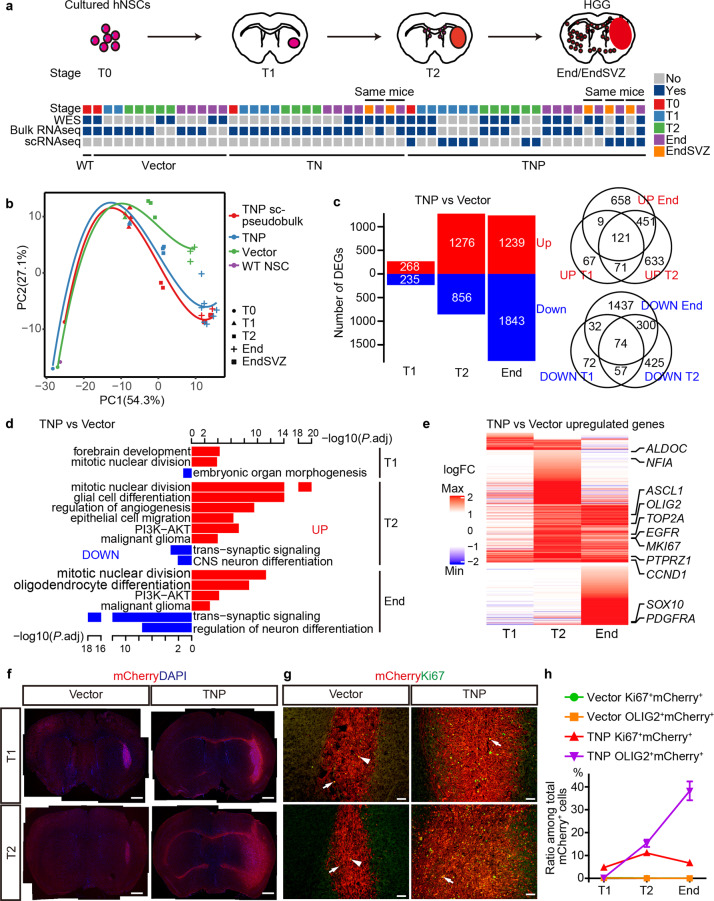
T2 represents an important stage of divergence between tumorigenesis and normal differentiation. **a** Scheme for sample collection of wild type (WT), Vector, TN, and TNP groups at four stages for WES, bulk RNA-seq, and scRNA-seq. **b** Principle component analysis (PCA) of bulk RNA-seq and single-cell pseudo-bulk of WT, Vector, and TNP samples at different stages. Dots and curves are colored by sample groups. Shapes represent different stages. **c** Left: The number of DEGs in TNP vs Vector at T1, T2, and End. Right: Venn diagrams illustrating the relationship of significantly upregulated (top) or downregulated (bottom) genes in TNP vs Vector at each stage. **d** Gene ontology enrichment analyses of DEGs in TNP vs Vector at each stage showing representative terms and adjusted *P* values (–log_10_). **e** Heatmap of the log_2_ fold-change for significantly upregulated genes in TNP vs Vector at each stage. **f** Low magnification view of mCherry^+^ DAPI^+^ cells in Vector and TNP brains at T1 and T2. Scale bars, 1 mm. **g** IF for mCherry/Ki67 in the central regions of Vector and TNP cells at T1 and T2. Triangular arrowhead, autofluorescent debris without DAPI staining. Arrows, co-labeled cells. Scale bars, 100 μm. **h** The ratio of Ki67^+^mCherry^+^ cells and OLIG2^+^mCherry^+^cells among total mCherry^+^ cells in Vector and TNP brains at each stage.

To delineate gliomagenic trajectories from mutant hNSCs to malignant gliomas in vivo, we first designated four stages during tumorigenesis and paralleled normal differentiation. In addition to the starting hNSCs, end-stage tumor and tumor-infiltrated SVZ samples (defined as stages T0, End, and EndSVZ, respectively), we further collected tissue from mice sacrificed one or two months post transplantation as early-stage samples (defined as stages T1 and T2, respectively), given that TN and TNP mice succumb to brain tumors as early as three months post transplantation. We then performed time-series bulk RNA-seq, scRNA-seq, and WES analyses in TNP, TN, Vector and wildtype hNSC samples at comparable stages (Fig. 3a).

To identify the divergent stage(s) between tumorigenesis and normal differentiation trajectories, we first compared the RNA-seq data from bulk and single-cell pseudo-bulk TNP samples to the controls. We used principle component analysis (PCA) and revealed that TNP and Vector samples were consistently separated by four different stages along the PC1 axis (Fig. 3b). While TNP samples cluster together with controls at T0 and T1, they started to diverge from controls at T2, and the difference became more evident at End/EndSVZ. This pattern was further confirmed by TNP pseudo-bulk samples compiled from scRNA-seq, which followed a similar trajectory (Fig. 3b).

To further assess the validity of the T2 as a divergent point and identify its enriched regulatory programs, we performed differential gene expression analysis on TNP and Vector samples at in vivo stages T1, T2, and End. Consistent with our PCA results, we identified 4.8 times and 3.6 times more upregulated and downregulated genes at T2 than T1, respectively (Fig. 3c; Supplementary information, Table S1). Differentially expressed genes (DEGs) at T2 were enriched for molecular functions and biological processes consistent with the functional differences between tumorigenesis and normal differentiation (Supplementary information, Table S2). 38 upregulated genes at this stage were enriched for malignant glioma (adjusted *P* value = 0.0001), including *OLIG2*, *EGFR*, *ASCL1*, *PTPRZ1*, and *CCND1* (Fig. 3d). Furthermore, many cancer hallmark pathways were upregulated at this stage, such as PI3K−AKT signaling, angiogenesis/vasculogenesis, and epithelial cell migration, which were consistently enriched at End but not T1 (Fig. 3d). Mitosis-related genes (e.g., *MKi67* and *TOP2A*) were more upregulated at T2 than End or T1, indicating that TNP cells at T2 had the greatest proliferation capacity (Fig. 3e; Supplementary information, Fig. S3a). In addition, glial differentiation pathways were also upregulated at T2 and End, suggesting that tumorigenesis co-opts but hyperactivates gliogenic programs of normal differentiation (Fig. 3d). Interestingly, astrocyte lineage genes (e.g., *NFIA* and *ALDOC*) were more upregulated at T2, while oligodendrocyte lineage genes (e.g., *PDGFRA* and *SOX10*) were more upregulated at End, suggesting stage-specific upregulation of lineage programs (Fig. 3e; Supplementary information, Fig. S3a). Consistent with the glial nature of malignant gliomas, genes downregulated at T2 and End were enriched for neuronal differentiation and synaptic functions (e.g., *DCX*, *NEUROD2*, and *SYN1*) (Fig. 3d).

To test whether such transcriptional dynamics are conserved across different glioma models, we repeated our analyses on bulk RNA-seq from TN and control samples, and observed similar patterns of the tumorigenic trajectory and differential gene expression burst at T2 stage that also enriched in malignant glioma-related pathways (Supplementary information, Fig. S3b–d, Tables S1, S2). Together, these results demonstrate that our models resemble the gliomagenesis process and identify stage-specific regulatory programs, highlighting T2 as a divergent point between tumorigenesis and normal differentiation characterized by a burst of oncogenic alterations.

To confirm these findings in vivo, we performed histological analyses on Vector and TNP samples at T1 and T2. Vector cells at both stages were mostly confined within the transplantation area with limited Ki67 or OLIG2 expression, and downregulated stemness marker hNES at T2 (Fig. 3f, g; Supplementary information, Fig. S3e, f). In contrast, TNP cells were locally expanded at T1, with very few mCherry^+^DAPI^+^ cells in the white matter or SVZ which did not express hNES (Supplementary information, Fig. S3g). At T2, TNP cells were dramatically expanded while maintaining their hNES expression (Fig. 3f; Supplementary information, Fig. S3f). A large number of mCherry^+^hNES^+^ cells diffusely infiltrated distant areas of the brain including bilateral SVZs, exhibiting histological features characteristic of malignant gliomas (Supplementary information, Fig. S3g). In addition, the frequency of cells expressing Ki67, OLIG2 at different stages is consistent with the transcriptional dynamics of these genes, peaking at T2 and End, respectively (Figs. 3g, h, 1f; Supplementary information, Fig. S3e). Thus, these observations validate our in silico findings and further support that T2 is an important stage during tumorigenesis.

### A persistent stem-like cell population at all stages of tumorigenesis

**Fig. 4:**
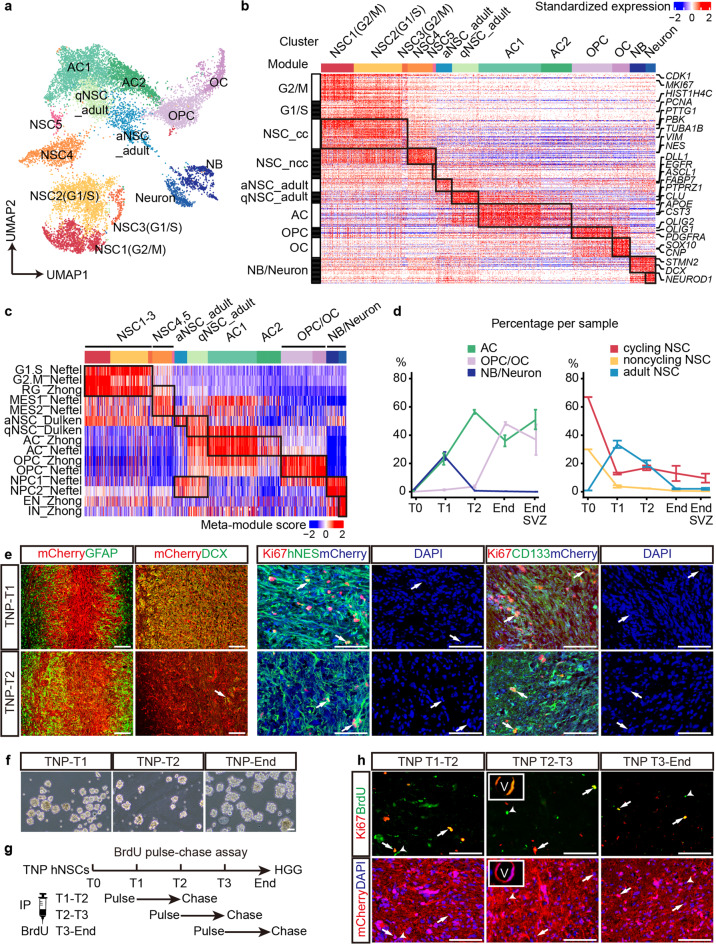
Time-series scRNA-seq uncovers temporal dynamics of distinct cell lineages. **a** UMAP analysis integrating high-quality single cells from TNP samples at T0 to End/EndSVZ. Clusters are separated by colors. **b** Heatmap of all single cells ordered by UMAP clusters. Columns, individual cells. Rows, genes. Boxed areas, gene expression modules. **c** Heatmap of scaled meta-module scores for each cluster based on published datasets. **d** The cellular frequencies of NSC, AC, OPC/OC, and NB/Neuron subpopulations per sample at different stages. Error bars, means ± SEM. **e** IF co-labeling of mCherry with Ki67/hNES, Ki67/CD133, GFAP and DCX in the central regions of TNP cells at T1 and T2. Arrows, triple- or double-positive cells. Scale bars, 100 μm. **f** Dissociated mCherry^+^ tissue samples from TNP mice at T1, T2, and End form spheres under the non-adherent stem cell culture condition. Scale bar, 100 μm. **g** The design and timeframe of BrdU pulse-chase assay. IP, intraperitoneal injection. **h** IF co-labeling of mCherry with Ki67/BrdU in the central regions of TNP cells from mice pulsed at T1, T2, and T3 and analyzed at T2, T3, and End, respectively. Arrows, BrdU^+^Ki67^+^mCherry^+^ cells localizing with DAPI. Arrowheads, BrdU^+^Ki67^–^mCherry^+^ cells. Insets, BrdU^+^Ki67^+^mCherry^+^ cells surrounding the blood vessel. V, blood vessel. Scale bars, 100 μm.

To further dissect the intra-tumor heterogeneity and pinpoint key cellular component(s) during gliomagenesis, we performed time-series single-cell transcriptomic analyses on the more aggressive and 100%-penetrant TNP model (Fig. 3a). Using the 10× Genomics platform, we sequenced and filtered cells based on stringent criteria (Materials and Methods), resulting in 13,642 high-quality human cells at four time points from T0 to End/EndSVZ TNP samples (*n* = 11 with biological replicates). Gene expression matrixes were processed using Harmony to minimize batch effects.^30^ We visualized all cells with Uniform Manifold Approximation and Projection (UMAP) and grouped them with unbiased graph-based clustering (Fig. 4a).^31^ The resultant 13 clusters expressed distinct lineage markers (e.g., *NES*, *ASCL1*, *PTPRZ1*, *APOE*, *OLIG2*, and *DCX)*, resembling human embryonic NSCs/radial glia (NSC1-5), astrocytes (AC1 and AC2), OPCs and oligodendrocytes (OPC and OC), neuroblasts and neurons (NB and Neuron), as well as quiescent and active NSCs in the adult mouse brain (qNSC_adult and aNSC_adult) (Fig. 4b, c). Embryonic NSC-like cells could be further divided into cycling (NSC1-3) and non-cycling NSCs (NSC4, 5), depending on their expression of cell cycle modules G1/S and G2/M (Fig. 4b, c). Based on these analyses, we defined eight cell-type-specific gene expression modules excluding G2/M and G1/S genes, namely NSC_cycling (NSC_cc), NSC_noncycling (NSC_ncc), aNSC_adult, qNSC_adult, AC, OPC, OC and NB/Neuron (Fig. 4b; Supplementary information, Table S3).

The subpopulation identities of all cells were further confirmed by meta-module score comparison using public human and mouse single-cell gene sets of developing/adult brains and GBMs (Fig. 4c).^5,32,33^ Consistent with previous reports, astrocytes and qNSCs share many gene expression patterns,^33^ and the NPC2 module by Neftel et al. more specifically marks the neuronal lineage than the NPC1 module, which is also expressed in oligodendrocytes (Fig. 4c).^5^

Next, we assessed the subpopulation dynamics during tumorigenesis (Materials and Methods). For the more differentiated lineages, we observed that neuron, astrocyte, and oligodendrocyte lineages reached their highest cellular frequency in a sequential manner, peaking at T1, T2, and End/EndSVZ, respectively. Immunostaining of lineage markers DCX, GFAP, and OLIG2 further supports a stepwise generation of each lineage (Fig. 4d, e, 3 h). Of note, the cellular composition of End and EndSVZ samples were mostly similar, except that EndSVZ samples had higher astrocyte frequency. For the stem-like lineages, over 97% of the TNP cells at T0 were cycling and non-cycling NSCs (Fig. 4e). As transplanted hNSCs generated more differentiated lineages at T1, both cycling and non-cycling NSCs dropped sharply, while adult NSCs cells reached their peaks. Strikingly, while non-cycling NSCs and adult NSCs reduced to below 2% at End, cycling NSCs remained stable at approximately 11%–17% from T1 till End, despite the dramatic expansion of TNP cells (Fig. 4e). These data indicate that cycling NSC-like cells are the major persistent stem-like population during gliomagenesis.

To identify these NSC-like cells in vivo, we used Ki67 as a surrogate marker since it was specifically expressed in cycling NSCs (Fig. 4c). Indeed, the majority of Ki67^+^ cells in TNP brains expressed stemness markers hNES and CD133 at both T1 and T2 (Fig. 4e). To functionally validate the presence of NSC-like cells, we dissected out mCherry^+^ regions from TNP mice at different stages (n ≥ 2 each), and cultured dissociated cells under the non-adherent stem cell culture condition. Cells from all of the samples formed spheres (8/8), similar to normal NSCs and GSCs (Fig. 4f). BrdU pulse-chase experiments have been commonly used for lineage-tracing of proliferating cells and identifying slowly-dividing, long-term BrdU label-retaining stem cells in vivo. We performed a single-day, five-time BrdU pulse on TNP mice at T1, T2, and T3 (around 3 months post transplantation) and analyzed the mice four weeks later or till End stage (Fig. 4g and Materials and Methods). We consistently identified BrdU^+^Ki67^+^mCherry^+^ long-term label-retaining cells at around 10% of total BrdU^+^mCherry^+^ cells (Fig. 4h). A subset of the label-retaining cells surround the blood vessels, reminiscent of GSCs (Fig. 4h). Together, these data support the persistence of a stem-like cell population at all stages.

To rigorously test these findings, we further analyzed cells by cluster and stage, and observed very consistent patterns (Supplementary information, Fig. S4a, b). To rule out the influence of cell cycle variances, we performed a simple linear regression against the cell cycle score in Seurat,^34^ and obtained similar results, confirming the cluster identity, lineage dynamics and the persistence of NSC-like cells (Supplementary information, Fig. S4c–f).

### De novo gliomagenesis is driven by sequential fate-switches of the NSC-like cells

**Fig. 5:**
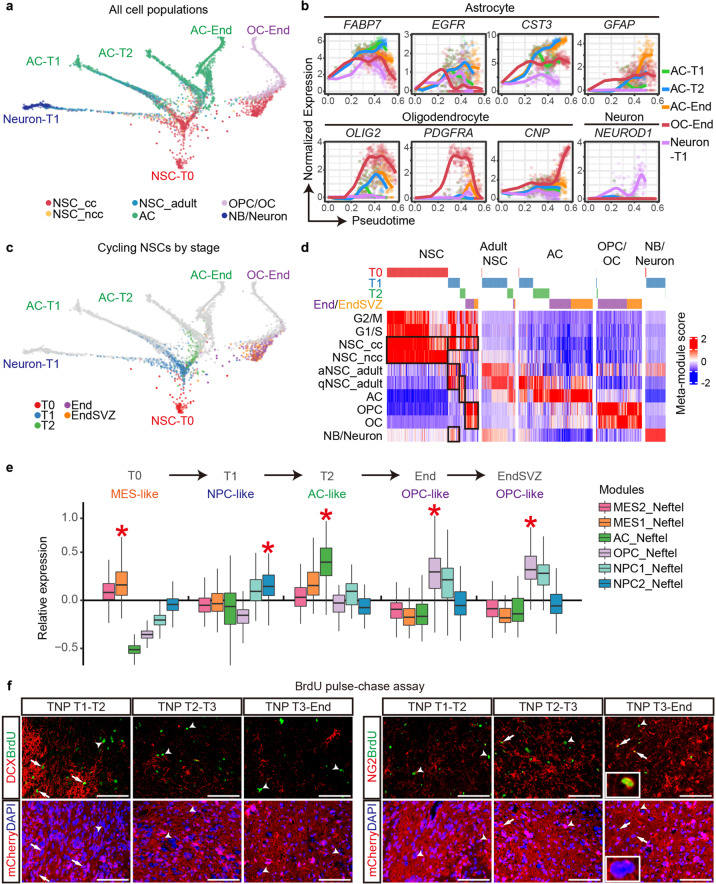
Reconstructed gliomagenesis tree highlights the fate switches of the NSC-like cells. **a** Gliomagenesis tree reconstructed by URD. Each subpopulation is separated by colors. **b** Pseudotime analyses for normalized expression of marker genes along different lineage trajectories. **c** Cycling NSCs at different stages highlighted on the gliomagenesis tree. **d** Heatmap of scaled meta-module scores for each subpopulation ordered by stage. **e** Relative expression of meta-modules by Neftel et al. in NSC-like cells at different stages of tumorigenesis. *, the most highly expressed module with statistical significance (*P* < 0.01). **f** IF co-labeling of mCherry with DCX/BrdU (left) and NG2/BrdU (right) in the central regions of TNP cells from mice pulsed at T1, T2, and T3 and analyzed at T2, T3, and End, respectively. Arrows, triple-positive cells. Arrowheads, BrdU^+^DCX^–^mCherry^+^ cells (left) or BrdU^+^NG2^–^mCherry^+^ cells (right). Insets, a representative BrdU^+^NG2^+^mCherry^+^ cell. Scale bars, 100 μm.

The unique lineage dynamics during gliomagenesis prompted us to investigate whether NSC-like cells sequentially generate more differentiated lineages. We first visualized all cells with a diffusion map by subpopulation and stage (Supplementary information, Fig. S5a, b). We observed six distinct tips with NSC at one end and more differentiated cells forming five distinct branching tips: neuronal cells at T1 (Neuron-T1), astrocyte from T1 to End/EndSVZ (AC-T1, AC-T2, and AC-End), and oligodendrocytes at End (OC-End). We further reconstructed a gliomagenesis tree using a diffusion-based simulation approach, URD.^35^ It inferred the pseudotime of each cell and performed random walks from the root (NSC-T0) to these tips to reconstruct a branching trajectory tree, which was visualized by a force-directed layout (Fig. 5a). The reconstructed tree largely resembled the lineage specification trajectories expected from classical neural differentiation models, based on the trajectory-specific expression of known marker genes (Fig. 5b; Supplementary information, Table S4).

Importantly, the gliomagenesis tree revealed the transcriptional dynamics of NSC-like cells during tumorigenesis. NSC-like cells spontaneously clustered by stage and occupied distinct branch points leading to different lineages, indicating a dramatic fate-switch (Fig. 5c; Supplementary information, Fig. S5b). To gain an overview of the transcriptional profiles of NSC-like cells at different stages, we grouped all single cells by stage and cell type, and compared their expression of our meta-gene modules (Fig. 5d). While NSC-like cells maintained the expression of NSC_cc module at all stages, they sequentially upregulate aNSC/neuron, qNSC/AC, and OPC/OC modules at T1, T2, and End/EndSVZ, respectively. Notably, T2 is a stage when NSC-like cells downregulate neuronal programs and upregulate glial programs, reminiscent of the neurogenic-to-gliogenic switch during normal neural development. This temporal gene expression pattern was further validated using published meta-modules (Supplementary information, Fig. S5c). It is evident that during in vivo gliomagenesis from T0 to End/EndSVZ, NSC-like cells sequentially upregulated MES-like, NPC2-like, AC-like and OPC-like meta-modules defined in end-stage human GBMs^5^ (Fig. 5e). Concurrently, we observe concerted tumor subtype conversions among individual TNP samples at different stages, from Mesenchymal to Classical and Proneural (Supplementary information, Fig. S5d).

To test whether NSC-like cells undergo fate-switches in vivo, we first analyzed their expression of OLIG2. Consistent with a neurogenic-to-gliogenic switch, mCherry^+^Ki67^+^ cells barely express OLIG2 at T1, yet the ratio of triple-labeled cells increased to around 40%–50% at T2 and 70%–80% at End (Supplementary information, Fig. S5e, f). Next, we quantified the lineage distribution of BrdU-labeled cells in the BrdU pulse-chase assay. We used DCX, GFAP, and NG2 as lineage markers for NPCs/neurons, astrocytes, and OPCs, respectively. We used stringent criteria to define co-localizing cells as the ones with mCherry^+^BrdU^+^ nuclei closely surrounded by the cytoplasmic staining of DCX, GFAP, or NG2. The majority of T1-labeled cells generated DCX^+^ cells, while T2-labeled cell predominantly generated GFAP^+^ cells (Fig. 5f; Supplementary information, Fig. S5g, h). The proportion of NG2^+^ cells increased from less than 1% during T1-T2 to 15%-18% during T3-End (Fig. 5f; Supplementary information, Fig. S5h). These data provide strong support fate-switches of NSC-like cells in vivo.

To test whether the fate-switch of NSC-like cells during gliomagenesis is dependent on the genetic context of TNP hNSCs, we further analyzed the TN samples. Deconvolution of TN bulk RNA-seq revealed a similar shift in cellular composition from neuronal dominance to glial dominance, and a late-stage emergence of the oligodendrocyte lineage (Supplementary information, Fig. S6a). Primary culture of TN tissue at different stages also generated spheres under the non-adherent stem cell culture condition (Supplementary information, Fig. S6b). RNA-seq of TN spheres at each stage further demonstrate their lineage bias towards neurons at T1 and astrocyte or oligodendrocyte at later stages (Supplementary information, Fig. S6c). Like in TNP mice, the ratio of Ki67^+^OLIG2^+^mCherry^+^ cells in TN mice significantly increased from T1 and T2 (Supplementary information, Fig. S6d, e). In addition, we generated another model (TP) using gRNAs targeting *TP53* and *PTEN* (Supplementary information, Fig. S6f). Of note, human GBM samples with mutations or deletions in both *TP53* and *PTEN* represent ~15% of TCGA GBM cohort. Although TP mice had a lower malignant glioma penetrance, we observed a significant decrease of Ki67^+^DCX^+^mCherry^+^ cells and an increase of Ki67^+^OLIG2^+^mCherry^+^ cells among total Ki67^+^mCherry^+^ cells from T1 to T2 (Supplementary information, Fig. S6g–i). Thus, the fate-switches of NSC-like cells during gliomagenesis do not appear to depend on the TNP genotype.

### Additional genetic alterations play a limited role in the sequential fate-switches of NSC-like cells

Genetic drivers such as *EGFR* and *PDGFRA* were proposed to influence the distribution of cellular states in human GBM.^5^ Thus, we sought to determine genetic alterations that drive the fate switches of NSC-like cells by analyzing the time-series deep WES (400X) data from TNP, TN, and Vector samples (Fig. 3a). The overall frequency of non-silent mutations in TN and TNP samples steadily increased from stages T1, T2 to End, reaching a level between human LGGs and GBMs (Supplementary information, Fig. S7a). We next assessed the recurrent mutations and did not detect clonal-level mutations among the 66 most frequently mutated genes in human GBM^36^ (Supplementary information, Fig. S7b), or any significant chromosomal alterations in WES-based chromosome number variation (CNV) analysis (Supplementary information, Fig. S7c). A small number of recurrently mutated genes, such as *SLC25A39* and *ERCC5*, had low clonal frequencies (below 5%) (Supplementary information, Fig. S7d), did not exhibit stage-specific enrichment, and were not previously shown to regulate the fate specification of NSCs.

Conversely, we consistently identified mutations or large deletions in the tumor-initiating drivers (*TP5*3/*NF1* and *TP5*3/*NF1*/*PTEN*) among TN and TNP samples, confirming the success of genome-editing. Provided that we did not perform single-cell colony selection, we reasoned that mosaic mutations co-existed in the initial TN and TNP hNSCs, which could be used as clonal lineage tracers. Indeed, we observed clonal selection patterns at the genome-edited loci of *TP53*, *NF1*, *PTEN*, and the selection at *TP53* loci was the most dramatic. It appears that TNP and TN tumors actively selected for *TP53* mutants with large deletion(s) between exons 5 and 6, evidenced by a progressive reduction of sequencing reads between the two *TP53* gRNA target sites (Supplementary information, Fig. S7e, f). Another example is a mutant form of *TP53* with a short deletion in exon 5, which was below the detection level at early stages but enriched in end-stage TNP samples (Supplementary information, Fig. S7g). Together, these data support that while TNP and TN hNSCs undergo clonal evolution and mutagenesis during tumorigenesis, additional genetic alterations play a limited role in the fate-switches of NSC-like cells in our models.

### The tumorigenic trajectory of NSC-like cells reveals stage-specific regulatory programs and resembles human GBM development

**Fig. 6:**
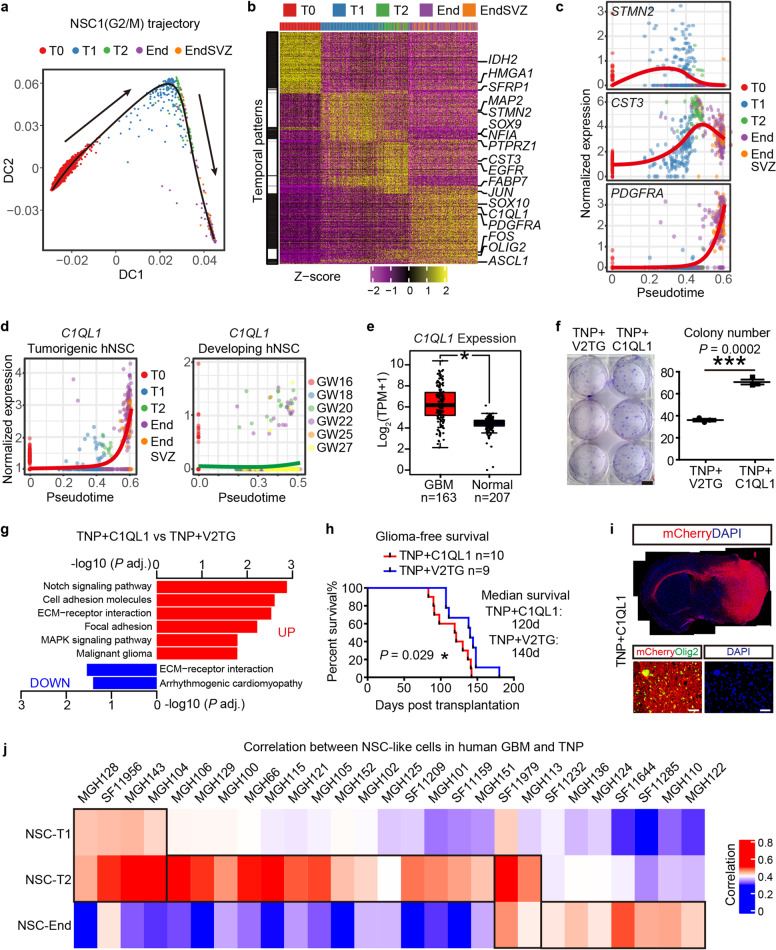
Pseudotime analyses identify stage-specific regulatory programs. **a** Diffusion map for NSC1 (G2/M), cells colored by stage. **b** Heatmap of gene expression cascades along the pseudotime of NSC1. Columns, pseudotime-ordered cells. Rows, genes. Colors in the top row indicate the actual sample time of each cell. Genes were clustered by their temporal expression patterns (left bars). **c** Trajectory plots of representative genes in (**b**) visualized by normalized expression. **d** Trajectory plots of *C1QL1* in tumorigenic TNP hNSCs (left) and embryonic hNSCs during human hippocampal development (right). **e** The log_2_-transformed TPM of *C1QL1* from TCGA GBM and matched GTEx normal brain RNA-seq. *P* < 0.01, one-way ANOVA test. **f** Left, colony formation assays of TNP + C1QL1 hNSCs and TNP + V2TG hNSCs. Scale bars, 1 cm. Right, the total number of colonies. *n* = 3 for each group. *P* values, Student’s *t*-test. **g** Gene ontology enrichment analyses of DEGs in TNP + C1QL1 vs TNP + V2TG hNSCs showing representative terms and adjusted *P* values (–log_10_). **h** The brain-tumor-free survival curves of TNP + C1QL1 and TNP + V2TG mice. n, the number of samples; *P* value, Log-rank (Mantel-cox) test. **i** IF for mCherry/DAPI and mCherry/OLIG2 in TNP + C1QL1 brains at the end stage. Scale bars, 1 mm (upper panels) and 50 μm (lower panels). **j** Heatmap of the Pearson correlation coefficients between NSC-like cells in human GBM samples (columns) and TNP samples at different stages (rows) based on their expression of DEGs in NSC1.

Since we did not observe additional fate-specifying genetic events, we further investigated whether the sequential fate switches of NSC-like cells were regulated through transcriptional reprogramming. We focused on the two major cycling NSC populations (NSC1 G2/M and NSC2 G1/S) and performed pseudotime analyses. The inferred pseudotime for these two populations largely resembled the actual time from T0 to End/EndSVZ (Fig. 6a, b; Supplementary information, Fig. S8a). Next, we determined stage-specific gene expression dynamics and categorized DEGs into distinct temporal patterns (Fig. 6b; Supplementary information, Fig. S8a and Table S5). We also performed differential gene expression analyses on cycling NSC-like cells in different branches to uncover the stage- and lineage-specific genetic guidance underlying gliomagenesis (Supplementary information, Fig. S8b, Table S6). Consistent with the sequential fate-switches of NSC-like cells, the expression of lineage genes for neurons, astrocytes and oligodendrocytes peaked at T1 (e.g., *MAP2*, *STMN2*), T2 (e.g., *CST3*, *SPARCL1, EGFR*) and End/EndSVZ (e.g., *PDGFRA, SOX10*), respectively (Fig. 6b, c). In addition to known markers, pseudotime analysis also identified more than 30 novel potential regulators among the top 100 DEGs, which have not been previously shown to drive gliomagenesis. For example, while many neuron-related genes were downregulated at stage End, *C1QL1*, a secreted protein implicated in synaptogenesis,^37^ was upregulated in tumorigenic TNP hNSCs (Fig. 6d). In contrast, we did not observe an upregulation of *C1QL1* in HOPX+ embryonic hNSCs during human hippocampal development, which also underwent neurogenic-to-gliogenic switch,^38^ suggesting that the upregulation of *C1QL1* is tumor-specific and may promote tumorigenesis (Fig. 6d). Consistently, *C1QL1* is expressed at a higher level in GBM compared to the normal brain using bulk RNA-seq data from TCGA and GTEx (Fig. 6e).

We further validated the functional significance of C1QL1 in gliomagenesis. When overexpressed in TNP hNSCs, *C1QL1* increased their colony formation capacity (Fig. 6f). RNA-seq analyses revealed that *C1QL1*-overexpression leads to elevated level of Notch signaling (a well-established stemness pathway)^39^ in TNP hNSCs (Fig. 6g). To examine the effect of *C1QL1* in vivo, we transplanted *C1QL1*-overexpressing TNP hNSCs into NOD-SCID mice. These mice developed malignant gliomas significantly faster than control TNP mice, with a median survival of 120 days compared to 140 days for controls (Fig. 6h, i). These data confirm that C1QL1 is a glioma promoting factor. Thus, pseudotime analyses identified stage-specific transcriptional programs, and uncovered novel oncogenic candidates for functional validations.

The plasticity of NSC-like cells is reminiscent of GSCs or stem-like cells in human gliomas. Thus, we investigated whether previously defined GSC signature genes were upregulated along our simulated tumorigenic trajectory of NSC-like cells. We compared the expression patterns of four published gene sets, including two gene sets determined by *IDH*-wildtype GBM cellular models (GBM GSC core TF and GBM stemness score), along with two stemness gene sets based on single-cell transcriptomic analyses of oligodendrogliomas and *IDH*-mutant gliomas^8,40–42^ (Supplementary information, Fig. S8c). In agreement with an NSC-to-GSC-like trajectory, GBM GSC core TF gene set was continuously upregulated along the pseudotime. Interestingly, GBM stemness score exhibited a different temporal pattern. While this gene set was consistently upregulated at in vivo stages T1 to End/EndSVZ compared to T0, it reached its peak at T2 instead of End/EndSVZ, suggesting that NSC-like cells already adopted a GSC-like “stemness” program before the development of full-blown tumors, consistent with the notion that T2 is an important stage for gliomagenesis. In contrast, the oligodendroglioma stemness score was not dramatically different at each stage, while the *IDH*-mutant glioma stemness score was reduced along the pseudotime, suggesting that our inferred pseudotime closely resembles the evolution of mutant NSCs towards GSCs of *IDH*-wildtype GBMs.

We further cross-examined 10X scRNA-seq data of both pediatric and adult GBMs with our gene modules,^5–7^ and identified similar NSC-like cells with strong NSC_cc signatures (Supplementary information, Fig. S8d, e). Importantly, NSC-like cells from individual patients can be divided into T1/T2-like, T2-like, T2/End-like, and End-like based on their correlation with NSC-like cells in our model at different stages, suggesting that gliomagenesis in patients may follow a similar trajectory of sequential fate-switches (Fig. 6j).

### The gliomagenic trajectory highlights stage-specific TF networks and provides a blueprint for early-stage interventions

**Fig. 7:**
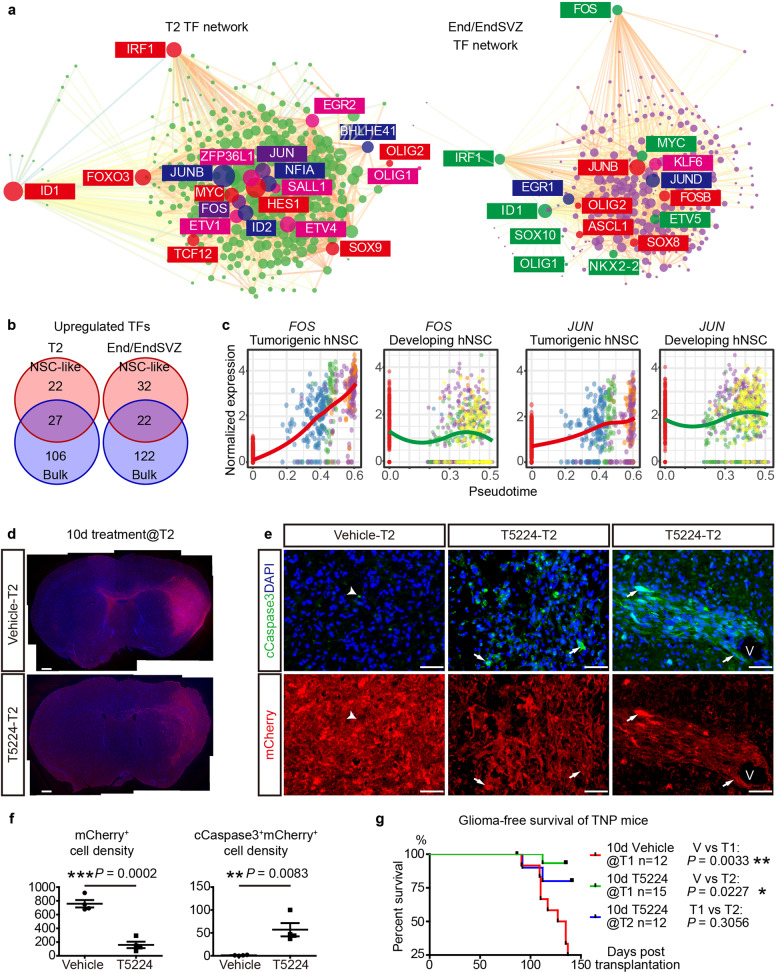
Transient early-stage AP-1 inhibition impedes gliomagenesis in vivo. **a** Stage-specific TF network in TNP NSC-like cells at T2 (left) and End (right). **b** The relationship between upregulated TFs in NSC-like cells at T2, End/EndSVZ from scRNA-seq, and those upregulated at the same stages in bulk TNP vs Vector samples (Fig. 3e). **c** Trajectory plots of *FOS* and *JUN* in tumorigenic TNP hNSCs (left) and developing embryonic hNSCs (right). **d** Low magnification view of mCherry^+^DAPI^+^ cells in brain sections of TNP mice treated with vehicle or T5224 for 10 days at T2. Scale bars, 1 mm. **e** IF of cleaved caspase 3 (cCaspase3) and mCherry on brain sections of T2 vehicle- or T5224-treated TNP mice. Arrows, co-labeled cells. Arrowhead, cCaspase3^+^/DAPI^–^ cell. V, blood vessel. Scale bars, 50 μm. **f** The number of mCherry^+^ and cCaspase3^+^mCherry^+^ cells per high-power field in the central tumor areas of TNP mice treated with vehicle or T5224 at T2 for 10 days. *n* = 4 for each group, *P* values from Student’s *t*-test. **g** The glioma-free survival curves of TNP mice treated with vehicle or T5224 at T1 or T2. n, the number of animals. *P* value, Log-rank (Mantel-cox) test.

Since NSC-like cells upregulated GBM core TFs, we sought to determine whether transcriptional reprogramming through TFs underlies the sequential fate switches of NSC-like cells. Indeed, many important TFs were upregulated at each stage and formed stage-specific transcriptional networks (Supplementary information, Table S5). While NSC-like cells at T2 and End/EndSVZ share a set of TFs at the core of the transcriptional network such as AP-1 TFs (*FOS*, *FOSB*, *JUN*, *JUNB*, *JUND*) and *MYC*, they respectively upregulate astrocyte-specifying TFs (e.g., *NFIA, SOX9*, and *HES1*) at T2 and oligodendrocyte-specifying TFs at End/EndSVZ (e.g., *NKX2-2* and *SOX10*) (Fig. 7a).^43^ Notably, unlike other oligodendrocyte-specifying TFs which were specifically upregulated at End/EndSVZ, *OLIG2* was already upregulated at T2, consistent with histological analyses (Supplementary information, Figs. S9a, S5e, f).

Intersectional analyses revealed that 40%–50% of upregulated TFs in NSC-like cells were not differentially expressed between bulk TNP and Vector samples at the same stages, indicating a cell-type-specific regulatory program in NSC-like cells (Fig. 7b). AP-1 TFs were among the TFs significantly upregulated in NSC-like cells but not in the bulk sample comparison. They occupied the center of the TF network at T2 and End, and were the top upregulated genes along the tumorigenic but not normal developmental trajectory (Fig. 7c). Thus, we sought to determine whether inhibition of AP-1 at different stages is sufficient to disrupt the tumorigenic trajectory of NSC-like cells. We used a specific AP-1 inhibitor T5224, which has advanced to phase II clinical trial but not been previously tested for the treatment of malignant gliomas.^44^ 10-day treatment at T2 dramatically reduced the area and density of TNP cells, and restricted their invasion (4/4) (Fig. 7d). Moreover, a significant portion of the remaining TNP cells underwent apoptosis characterized by high-level expression of cleaved Caspase 3, which rarely occurs in vehicle-treated TNP mice (Fig. 7e, f). Apoptotic cells were particularly evident around the blood vessels, where many Ki67^+^ NSC-like cells reside (Fig. 7e). Consistently, we observed a drastic decrease of Ki67^+^ cells and OLIG2^+^ cells in the treatment group (Supplementary information, Fig. S9b). We repeated this transient 10-day T5224 treatment paradigm on TNP mice at T1 and T2 based on a larger cohort of >10 mice in each group, and observed significant survival benefits (Fig. 7g).

In contrast, when we treated TNP mice at T3 with vehicle or T5224 for 10 days, mice from both groups still developed tumors as early as 20 days after treatment withdrawal, even though we observed large necrotic regions in the tumor areas of T5224-treated mice but not in vehicle-treated controls (Supplementary information, Fig. S9c, d). Taken together, these results support the notion that different TFs networks may drive the sequential fate switches of NSC-like cells, and targeting core TFs such as AP-1 at early stages of gliomagenesis may disrupt the tumorigenic trajectory of NSC-like cells.

## Discussion

The poor prognosis of GBM underscores the need to develop novel diagnostic and therapeutic paradigms based on a better understanding of the disease etiology. In this study, we traced the natural history of gliomagenesis in hNSC-derived malignant glioma models, and depicted a comprehensive, multi-omic landscape of de novo gliomagenesis, shedding light on an important yet understudied “black box” biological process.

Our analyses are mainly built upon two newly developed, highly-penetrant malignant glioma models that resemble human GBM, offering us a unique opportunity to track de novo gliomagenesis from hNSCs in vivo, which has not been achieved in previous NSC-derived glioma models. Since these models are developed from a well-characterized human ES cell line (Hues8), they are easily reproducible and can be scaled up for preclinical drug screening. By alternating the initiating mutations, our protocol would allow standardized and fast generation of a series of hNSC-derived, human-relevant glioma models. One limitation of these models is that tumorigenesis occur in an immunocompromised microenvironment. The impact of immune cells on the gliomagenic trajectory needs to be determined in humanized mouse models in future studies. In addition, unlike human tumors, CNVs are infrequent in these models, possibly due to the relatively short tumor latency and the introduction of strong genetic drivers reducing the selective pressure for CNV drivers.

The genetic combinations we used in this study are present in human GBMs,^3,20^ and our key findings are validated by human GBM samples and a TP model representing a more general genetic context. While we cannot rule out that hNSCs with different driver combinations or with a different genetic background may exhibit distinct tumorigenic trajectories, the consistency among our models and the fact NSC-like cells in human GBM align in a similar trajectory supports a common path of de novo tumorigenesis from mutant hNSCs.

We defined different stages of early gliomagenesis in vivo and identified a persistent, highly proliferative NSC-like subpopulation at all stages through scRNA-seq and BrdU label retention assays. Abnormal NSC/NPC-like cells at early stages of gliomagenesis have been observed in several GEMMs, and were proposed to drive tumor initiation and invasion.^10,14,45^ However, these studies lack the resolution to comprehensively characterize these cells and track their fate during early tumorigenesis. Through multi-omic time-series analyses, we revealed that hNSCs and NSC-like cells, even after acquiring all the necessary genetic drivers, need to undergo multi-step reprogramming and fate-switches to generate malignant lineages. These cells sequentially adopt NPC-like, AC-like, and OPC-like programs to generate distinct malignant lineages while maintaining NSC characteristics, which is confirmed by lineage tracing experiments. Importantly, NSC-like cells with each fate can be correlated with their counterparts in individual GBM samples, which validates the human relevance of our models. Furthermore, this may provide a potential explanation for a longstanding question in the field why GBMs from different patients have distinct types of lineage-biased glioma stem/stem-like cells.^6,46^

The fate-switch model also helps address how human GBMs end up with vastly different distributions of heterogeneous cellular states. Such distribution is likely determined by the specific stage when the tumor becomes clinically manifested along the tumorigenic trajectory (Supplementary information, Fig. S9e). This may be influenced by stage-specific fate determinants uncovered in this study, as well as known genetic drivers such as *EGFR* and *PDGFRA* amplification. Tumors manifested at intermediate stages could exhibit hybrid phenotypes, which were observed in human GBMs (Fig. 6j).^4,5^ Our model system would serve as a good platform to test these hypotheses in future studies.

While previous studies have drawn similarities between tumorigenesis and normal development, we rigorously distinguished these two trajectories. We show that glioma initiating cells at different stages do not bear the same level of oncogenic alterations, but rather exhibit an “oncogenic burst” during the neurogenic-to-gliogenic switch, characterized by an abrupt 5-fold increase of DEGs. TNP cells during the neurogenic phase are not dramatically different from control at the transcriptional level and pathologically mimic “carcinoma in situ”. Only after the neurogenic-to-gliogenic switch do they diffusely infiltrate the brain areas including the SVZ. Thus, the period during which hNSCs undergo neurogenic-to-gliogenic switch may represent a window of opportunity for early-stage diagnosis and treatment.

We functionally confirmed two actionable targets (C1QL1 and AP-1) based on our analyses of the gliomagenic trajectories. C1QL1 is one of the few neuron/synapse-related proteins that are upregulated in NSC-like cells, against the backdrop of a general downregulation of neuronal programs. While more detailed mechanism of its tumor-promoting function is yet to be determined, its reported role in synapse formation suggests it may be involved in the recently discovered glioma-neuron interactions.^47,48^ As a secreted protein, C1QL1 may also serve as an early diagnostic marker for GBM. As for AP-1 TFs, we show that they are the core TFs in the transcriptional network during the neurogenic-to-gliogenic switch, and transient AP-1 inhibition at this stage with T5224, a drug not previously used for the treatment of malignant glioma, is sufficient to inhibit gliomagenesis in vivo and provide survival benefits. Alternatively, targeting the fate-switch regulators could also potentially inhibit gliomagenesis. Thus, the tumorigenic trajectory reveals stage-specific treatment vulnerabilities in NSC-like cells and potential early diagnostic markers before the clinical manifestation of GBM.

In summary, our data demonstrate the critical steps and molecular regulators underlying de novo gliomagenesis, and provide a valuable resource and a blueprint for potential stage-specific early interventions to disrupt the tumorigenic trajectory.

## Materials and methods

### Cell lines and cell culture

The iCas9 hPSCs were gifted by Dr. Danwei Huangfu at Sloan-Kettering Institute and Dr. Jie Na at Tsinghua University. iCas9 hNSCs were differentiated from iCas9 hPSCs based on previous reports.^49^ Briefly, iCas9 hPSCs were cultured on matrigel-coated dishes and fed daily with mTeSR (STEMCELL) for 7 days. On the next day, mTeSR was substituted by N2 medium (DMEM/F12 supplemented with 0.5× N2 supplement (Gibco), 1 μM dorsomorphin (Tocris), and 1 μM SB431542 (STEMCELL)) for 1–2 days. hPSC colonies were lifted off, cultured in suspension on the shaker (95× rpm at 37 °C) for 8 days to form embryoid bodies (EBs) and fed with N2 media. EBs were then mechanically dissociated, plated on a matrigel-coated dish, and fed with hNSC maintenance medium (DMEM/F12 supplemented with 1× N2 supplement, 1× B27 supplement (Gibco), 1% penicillin/streptomycin, and 20 ng/mL bFGF (Gibco)). The emerging rosettes were picked manually, dissociated completely using Accutase (Gibco), and plated on a poly-ornithine/laminin-coated plate. The resultant hNSCs were expanded and maintained in the hNSC maintenance medium. The 293T cells were purchased from the cell resource center of Shanghai Institutes for Biological Sciences, Chinese Academy of Sciences and cultured in DMEM medium with 10% FBS and 1% penicillin/streptomycin (Gibco).

### Animals

Female NOD/SCID mice at 4–5 weeks of age were purchased from Beijing Vital River Laboratory Animal Technology (Beijing, China). Mice were housed in pressurized, individually ventilated cages (PIV/IVC) and maintained under specific-pathogen-free conditions, with free access to food and water in a 12 h light/dark cycle. All animal studies were approved by the Animal Care and Use Committee of Sichuan University.

### Orthotopic xenograft mouse models

Vector, TN, TNP, TP, TNP + V2TG, and TNP + C1QL1 hNSCs were dissociated by Accutase. Single-cell suspensions were prepared in sterile Hanks Balanced Salt Solution (HBSS, Gibco) immediately before the xenograft procedure. 1 × 10^5^ cells in 4 µL HBSS were stereotaxically injected into the striatum of female NOD/SCID mice at 5–6 weeks of age using a 10 μL micro-syringe (RWD life science, Shenzhen, China). Stereotactic coordinates used were 0.5 mm anterior to the bregma, 3.0 mm lateral to the midline, and 3.0 mm deep. After the infusion of cells, the syringe needle was kept in place for 2 min, and then withdrawn manually at a rate of 0.875 mm/min to minimize the backflow of cells.

### Vectors and gRNAs

All the gRNAs used in this study were designed at the ATUM website (https://www.atum.bio/eCommerce/cas9/input) and synthesized by TsingKe Biological Technology (Beijing, China). At least 3 top-ranked gRNAs were selected for each genetic locus. These gRNAs were cloned into pLentiCRISPR V2 (Addgene), and their targeting efficiency was assessed in 293T cells. The most efficient gRNAs evidenced by T7EI and western blot analyses were selected for subsequent genome-editing in iCas9 hNSCs and listed below:

*TP53*-5-gRNA (exon 5) caccgGGCACCCGCGTCCGCGCCA

*TP53*-6-gRNA (exon 6) caccgAACACTTTTCGACATAGTG

*NF1*-1-gRNA (exon 1) caccGGGAGGACATGGCCGCGCAC

*NF1*-31-gRNA (exon31) caccgACTGTAGCTTTATTCAGTA

*NF1*-32-gRNA (exon 32) caccgAGAACAGCATCGGTGCAGT

These gRNAs were cloned into pLentiV2T-mCherry (V2TC) vector, which was constructed from pLentiCRISPR V2 vector by replacing Cas9 sequence with a reporter gene mCherry. The gRNA combination for TN group includes gRNAs targeting *TP53* exons 5 and 6, *NF1* exons 31 and 32. The gRNA combination for TNP group includes gRNAs targeting *TP53* exons 5 and 6, *NF1* exon 1, and *PTEN* exon 1. The gRNA combination for TP group includes gRNAs targeting *TP53* exons 5 and 6, and *PTEN* exon1. Every gRNA is driven by a unique human U6 promoter. To overexpress genes in TNP hNSCs, we constructed pLentiV2TG vector from pLentiV2TC by changing the mCherry sequence to neomycin- and kanamycin-resistant genes NeoR/KanR, and replacing the sequence from hU6 promotor to U6 terminator with EF-1α-core promoter sequence. The coding sequence of *C1QL1* was synthesized by Sangon Biotech (Shanghai, China), and was cloned into pLentiV2TG downstream of the EF-1α-core promoter.

### Lentiviral packaging and viral transfection of hNSCs

Lentiviruses carrying V2TC, V2TC-TN, V2TC-TNP, V2TC-TP, V2TG, and V2TG-C1QL1 were produced in 293T cells through calcium phosphate precipitation packaging system. Harvested lentiviruses were further concentrated (10×) by Lenti-X™ Concentrator kit (Takara) to remove excessive FBS. For genome-editing in iCas9 hNSCs, cells were plated on 6-well plates coated with 10 μg/mL polyornithine (Sigma) and 5 μg/mL laminin (Gibco), and cultured in hNSC maintenance medium with 2 μg/mL doxycycline. Once hNSCs reached 50% confluency 12-24 h after being plated, 100 μL concentrated lentivirus carrying V2TC, V2TC-TN, V2TC-TNP, or V2TC-TP were added to the medium for 8 h. Infected hNSCs were cultured in fresh medium containing 2 μg/mL doxycycline for 48 h, and then screened in the culture medium containing 1 μg/mL puromycin. The resultant hNSCs were passaged three times, and the mutation efficiency was assessed by T7EI, quantitative real-time PCR (qRT-PCR) and western blot. To overexpress *C1QL1* in TNP hNSCs, hNSCs were similarly plated and infected with lentiviruses carrying V2TG or V2TG-C1QL1 for 8 h. Infected hNSCs were cultured in fresh medium for 48 h, and then screened in the culture medium containing 800 μg/mL G418 until positive colonies formed in about two weeks. The resultant hNSCs were passaged three times, and the expression levels of *C1QL1* in these cells were assessed by qRT-PCR.

### T7EI analysis for assessment of genome-editing efficiency

Genomic DNA was extracted from control and mutant hNSCs. Genomic regions flanking the gRNA-target sites were amplified by PCR. For T7EI assays, 5 μL of PCR products were denatured and re-annealed in T7EI Buffer (ViewSolid Biotech, Beijing) in a total volume of 10.5 μL using the following protocol: 95 °C, 5 min; 95 °C–75 °C at –0.1 °C/s; 75 °C–16 °C at −0.1 °C/s; 16 °C, 2 min. Then, 10.5 μL of hybridized PCR products were treated with 5U T7EI enzyme at 37 °C for 30 min in 11 μL final reaction volume. Products were then analyzed on 2% agarose gels and imaged with a Gel Doc imaging system (Bio-Rad). Quantification was based on relative band intensities measured by ImageJ. Indel percentage was determined by the formula 100 × (1– (1– (b + c) / (a + b + c))1/2), where a is the integrated intensity of the undigested PCR product, and b and c are the integrated intensities of each cleaved product.^50^ PCR primers are listed below:

*TP53*-p5F: 5’TGTAGACGCCAACTCTCTCT3’

*TP53*-p5R: 5’GCAATCAGTGAGGAATCAGAGG3’

*TP53*-p6F: 5’GCCTCTGATTCCTCACTGATT3’

*TP53*-p6R: 5’TTTCACCGTTAGCCAGGATG3’

*NF1*-p31F: 5’AGTAGACATGATTGGGTCTCAAC3’

*NF1*-p31R: 5’GTGACTCTTTCCCACCATATACTT3’

*NF1*-p32F: 5’ATTTGGTCTGCTTTCATTACTCATC3’

*NF1*-p32R: 5’GGTAGTGTTTCTAACCTTCCCA3’

*NF1*-p1F: 5’CGTGGAAAGGATCCCACTT3’

*NF1*-p1R: 5’GTTACCCACCTCTGCTCAAA3’

*PTEN*-p1F: 5’CAGCCGTTCGGAGGATTATT3’

*PTEN*-p1R: 5’CCCTCAGGAAGAGACCATATAGA3’

### Tissue preparation for histology and sequencing

For histological analysis, we utilized both paraffin and frozen sections. Mice at various time points were perfused with 4% paraformaldehyde (PFA). Brains were dissected, prepared as coronal brain slices, and processed for either paraffin-embedded or frozen sections. For paraffin sections, brains were post-fixed in 4% PFA overnight at 4 °C, and paraffin-embedded after dehydration (Leica). For frozen sections, brains were post-fixed in 4% PFA overnight at 4 °C, and then transferred to 30% sucrose overnight at 4 °C. Dehydrated brain tissues were then embedded in O.C.T. compound (Tissue-Tek) and frozen on dry ice. Serial sections were coronally prepared at 5 μm for paraffin sections or 10 μm for cryostat sections.

For genomic and transcriptomic analyses, mice at various time points were sacrificed by cervical dislocation. Brains were quickly dissected, washed twice with ice-cold DPBS (without Ca^2+^ and Mg^2+^, Gibco), and prepared as coronal brain slices. Tissues around the transplantation sites or along the lateral ventricle/SVZ with strong mCherry signals were carefully and maximally dissected under a fluorescent dissection microscope (Olympus). For bulk WES and RNA-seq, dissected tissues were snap-frozen in liquid nitrogen and sent to Novogene (Beijing, China) and Anoroad (Beijing, China) for DNA/RNA extraction and sequencing. For scRNA-seq, dissected tissues were cut into small pieces, and incubated with 1 mg/mL collagenase type I (Gibco) plus 0.5 mg/mL collagenase type IV (Gibco) at 37 °C for 15 min, followed by mechanical dissociation through pipetting for 10 times. Dissociated cells were filtered through a 70 μm strainer, and centrifuged at 300× g for 5 min. The resultant single cells were washed with HBSS w/o Ca^2+^ and Mg^2+^ (Gibco) for two times, and re-suspended as single cells at a concentration of 1.2 × 10^3^ cells/µL in HBSS w/o Ca^2+^ and Mg^2+^. For each sample, 10,000 cells (live cells > 90%) were used for subsequent library construction. For scRNA-seq on cultured TNP cells, cells were dissociated with Accutase, washed with HBSS w/o Ca^2+^ and Mg^2+^ for two times, and re-suspended as single cells at a concentration of 1 × 10^3^ cells/µL in HBSS w/o Ca^2+^ and Mg^2+^. 8000 cells (live cells > 90%) were used for subsequent library construction.

### Western blot analysis

Cells were harvested, washed with phosphate-buffered saline (PBS), lysed in RIPA buffer (Beyotime Biotechnology) with 1 mM PMSF, and centrifuged at 14,000× g, 4 °C for 5 min. Protein samples (approximately 20 μg each) were analyzed by SDS-PAGE and electro-transferred to a PVDF membrane (Millipore). The blots were then blocked in 5% non-fat milk in TBST, followed by incubation of primary antibodies at 4 °C overnight. After washing, the blots were incubated in horseradish peroxidase (HRP)-conjugated secondary antibodies at room temperature for 1 h. Signals were detected using ECL or ECL Plus (GE Healthcare) followed by film development. The primary antibodies used are as follows: Pten (1:1000, Cell Signaling Technology), Nf1 (1:1000, Abcam), p53 (1:1000, Abcam), β-Actin (1:1000, Cell Signaling Technology), GAPDH (1:1000, Cali-Bio).

### Immunofluorescence (IF)

For IF staining on tissues, frozen brain sections were oven-dried at 42 °C for 30 min, rinsed and rehydrated with PBS, and treated with 0.3% Triton X-100 in PBS for 20 min at RT. Sections were then blocked with 2% goat serum in PBS for one hour at RT, and incubated with primary antibodies overnight at 4 °C. Primary antibodies were visualized by species-specific goat secondary antibodies conjugated to Alexa Fluor dyes (Alexa 488/555/647, 1:1000, Invitrogen). Sections were then stained with DAPI (1 μg/mL) for 5 min. Slides were coverslipped and imaged under an Olympus BX51 fluorescent microscope. Antibodies used in this study were: Olig2 (1:1000, Millipore), GFAP (1:2000, Abcam), hNESTIN (1:1000, Abcam), Ki67 (1:200, BD), CD133 (1:1000, Abcam), CD31 (1:100, BD), Cleaved Caspase 3 (1:500, Cell Signaling Technology), αSMA (1:50, Abcam), mCherry (1:2000, Abcam), pERK (1:100, Cell Signaling Technology), DCX (1:1000, Abcam), NG2 (1:100, Millipore), BrdU (1:500, Abcam).

For IF staining of cultured hNSCs, cells were fixed with 4% PFA for 15 min at RT. After three washes with PBS, cells were treated with 0.5% Triton X-100 for 15 min at RT. After blocking with 5% milk in PBS for 1 hour at RT, cells were incubated with anti-hNESTIN (1:2000; Abcam), anti-Sox2 (1:1000; Abcam), anti-Pax6 (1:500; Abcam), anti-mCherry (1:2000; Abcam) overnight at 4 °C. Primary antibodies were visualized by species-specific goat secondary antibodies conjugated to Alexa Fluor dyes (Alexa 488/555/647, 1:1000, Invitrogen), and the nuclei were stained with DAPI (1 μg/mL). Stained cells were coverslipped and imaged under a Zeiss (LSM880) confocal microscope.

IF images presented in the figures are representative of at least three biological replicates in each group.

### Colony formation assay

To determine the colony-formation capacity of Vector, TN, and TNP hNSCs, 1500 cells for each group were seeded in the coated 6-well plate, and cultured until apparent colony formation. For TNP + V2TG and TNP + C1QL1 hNSCs, only 1000 cells were used for each group due to the high colony-formation capacity of TNP hNSCs. Colonies were stained by crystal violet (Beyotime biotechnology), and the total colony numbers were counted and compared.

### qRT-PCR

Briefly, total RNA was purified from hNSCs using TRIzol reagent (ThermoFisher Scientific). 2 μg RNA for each sample was reverse-transcribed into cDNA by FastKing-RT SuperMix Kit (Tiangen), prepared in iTaq™ Universal SYBR Green Supermix (BioRad), and analyzed by BioRad CFX96 Touch Real-Time PCR Detection System. The average threshold was determined for each gene and normalized to β-Actin or GAPDH. Primers used for qRT-PCR are listed below:

*C1QL1*-cDNA-F: 5’CATTCCCGGCACCTACTTT3’

*C1QL1*-cDNA-R: 5’GCCAGAGAACGTGCTGTATTT3’

*GAPDH*-cDNA-F: 5’GGAGCGAGATCCCTCCAAAAT3’

*GAPDH*-cDNA-R: 5’GGCTGTTGTCATACTTCTCATGG3’

*ACTIN*-beta-cDNA-F: 5’CATGTACGTTGCTATCCAGGC3’

*ACTIN*-beta-cDNA-R: 5’CTCCTTAATGTCACGCACGAT3’

### Primary sphere culture

To culture NSC-like cells from primary tissues of TNP and TN mice at various time points, tissues around the transplantation sites with strong mCherry signals were carefully and maximally excised from mouse brains under a fluorescent dissection microscope (Olympus). Dissected tissues were cut into small pieces, and incubated with 1 mg/mL collagenase type I (Gibco) plus 0.5 mg/mL collagenase type IV (Gibco) at 37 °C for 15 min, followed by mechanical dissociation through pipetting for 10 times. Dissociated cells were filtered through a 70 μm strainer, and centrifuged at 300× g for 5 min. The resultant single cells were washed with HBSS w/o Ca^2+^ and Mg^2+^ (Gibco) for two times, and non-adherently cultured in stem cell culture medium (Neurobasal Medium (Gibco) supplemented with GlutaMAX (Gibco) and Sodium Pyruvate (Gibco), 1× B-27 supplement, 20 ng/mL EGF, and 20 ng/mL bFGF) in 6-well ultra-low binding plates (Corning). Cells were grown until they form spheres for subsequent analyses. The images presented in the figures are representative of at least two biological replicates in each group.

### BrdU pulse-chase assay

TN and TNP mice at T1, T2 and T3 (around 3 months post transplantation) stages were pulsed with 50 mg/kg (gram, body weight) of BrdU five times daily at two-hour intervals. Mice pulsed at T1 and T2 were sacrificed four weeks after the initial pulse. Mice pulsed at T3 were aged until signs of distress appeared and sacrificed. BrdU immunofluorescence was performed as previously described on brain sections near the transplantation sites.^14^

### Administration of c-Fos/AP-1 inhibitor T5224

T5224 was dissolved in 10% polyvinylpyrrolidone (vehicle, Sigma). TNP mice at T1, T2, or T3 were randomly divided into two groups, and treated with vehicle or T5224 (120 mg/kg body weight) through oral administration for 10 days. To assess the effect of acute treatment, TNP mice treated at T2 with vehicle or T5224 (4 each) were sacrificed one day after the 10-day treatment. For survival curve comparison, mice were aged until signs of distress appeared, sacrificed, and subjected to histological assessment of glioma development.

### Bulk WES sequencing and analysis

#### Library preparation and sequencing

Genomic DNA was extracted from brain tissues by Blood & Tissue Genomic DNA Extraction Kit (Tiangen). Sequencing libraries were generated using Agilent SureSelect Human All Exon kit V6 (Agilent Technologies, CA, USA) following the manufacturer’s recommendations. Briefly, fragmentation was carried out by the hydrodynamic shearing system (Covaris, Massachusetts, USA) to generate short fragments. The sequencing libraries were constructed on a cBot Cluster Generation System using Hiseq PE Cluster Kit (Illumina) according to the manufacturer’s instructions. Libraries were subjected to 150 bp paired-end sequenced on the Illumina NovaSeq 6000. Each sample was sequenced to 400× coverage with an average of 161 million (M) reads (SD = 21 M).

#### Pre-alignment QC

Prior to alignment reads were initially subjected to a quality control step using FastQC (v0.10.1).^51^ Reads containing adapter, poly-N, and with low quality were removed to obtain the clean data, which were further filtered based on their Q20, Q30 and GC content to meet the standard (Q20 > 90, Q30 > 85) by Trimmomatic.^52^ All the downstream analyses were based on these clean data.

#### BAM processing

To call human genomic variants from WES data, all exome paired-end reads were aligned to the combined genome of hg19/GRCh37 (https://grch37.ensembl.org/Homo_sapiens) and mm10 (https://ensembl.org/Mus_musculus) using BWA (0.7.17-r1188)^53^ and sorted by sambamba (v0.6.6).^54^ The reads uniquely mapped to either the human or mouse genome were extracted into human and mouse bam files, respectively. The human bam files were then sorted, and only paired reads were kept. After marking duplicates, the paired-only bam files were processed using BaseRecalibrator and ApplyBQSR in GATK (v4.1.0).^55^ We then calculated the coverage of target regions provided by Agilent using CollectHsMetrics module in GATK. Only samples in which over 80% of targeted bases with at least 30× coverage were included for downstream analyses. All analysis procedures were integrated by Snakemake (v5.5.2).^56^

#### Somatic variant calling

Single nucleotide variants (SNPs) and insertions/deletions (Indels) were called using Mutect2^57^ in GATK. Somatic variants were called by comparing the corresponding tumor sample to its vector samples at the same time point. Given the CRISPR-induced mutations at the target sites occur randomly with varying frequency, we applied the filter criterion (-max-events-in-region 5 -max-alt-allele-count 2) to explore the clonal selection. For other somatic variants, we used the default parameters to filter the somatic variants. These variants were annotated by VEP (ensembl-vep-release-97.3).^58^ The downstream analyses and visualization were processed by R (3.6.0) package maftools (2.0.16).^59^

#### Copy number variation analysis

Significant focal somatic copy-number alterations (SCNAs) were inferred by CNVkit^60^ using Circular Binary Segmentation algorithm with default parameters.^61^ Segment-level ratios were calculated and log_2_ transformed. SCNAs across all samples were identified by Genomic Identification of Significant Targets in Cancer (GISTIC, version 2.0)^62^ to determine which SCNA regions were significantly gained or lost than expected by chance with *q* value ≤ 0.01.

#### Tumor mutational burden (TMB) analysis

To calculate the TMB, the total number of mutations counted was divided by the size of the target sequence region of the Agilent SureSelect Human All Exon V6. The TMB profiles of thymoma (THYM), brain lower-grade glioma (LGG), glioblastoma multiforme (GBM), and skin cutaneous melanoma (SKCM) in TCGA were downloaded from https://gdc.cancer.gov/about-data/publications/PanCan-CellOfOrigin.

### Bulk RNA sequencing and analysis

#### Library preparation and sequencing

Total RNA was purified using TRIzol reagent (Invitrogen). RNA purity was checked using the NanoPhotometer® spectrophotometer (IMPLEN, CA, USA). RNA concentration was measured using Qubit® 2.0 Fluorometer (Life Technologies, CA, USA). RNA integrity was assessed using the RNA Nano 6000 Assay Kit of the Bioanalyzer 2100 system (Agilent Technologies, CA, USA). Sequencing libraries were generated using NEB Next® UltraTM RNA Library Prep Kit for Illumina® (NEB, USA) following the manufacturer’s recommendations. The library fragments were purified with AMPure XP system (Beckman Counlter, Beverly, USA). The libraries were sequenced on the Illumina HiSeq 2500 platform and 150 bp paired-end reads were generated (Anoroad, Co, Ltd, Beijing, China). Each sample was sequenced to an average depth of 65 M reads (SD = 7 M).

#### QC and Alignment

Fastq files were initially subjected to a quality control step using FastQC (v0.10.1),^51^ and the reads were then trimmed using Trimmomatic. To accurately quantify human gene expression, we applied a two-step alignment. The filtered reads were first mapped to a combined reference genome from human (hg19) and mouse (mm10) using STAR (v2.7.1a).^63^ The reads that uniquely aligned to the human genome were extracted and converted into fastq format by sambamba. We then aligned the cleaned human reads to the hg19 genome for downstream analyses.

#### Differential expression and gene pattern analysis

For differential gene expression analysis, we used DESeq2 (v1.24.0)^64^ to perform normalization and differential expression test on the raw read counts for each gene annotated in GRCh37 (release 87 ftp.ensembl.org/pub/grch37/release-87/gtf/homo_sapiens/Homo_sapiens.GRCh37.87.gtf.gz). Differential expression genes (DEGs) were defined using DESeq2 with the adjusted *P* value < 0.05 and absolute log_2_(fold-change) > 1.2. The KEGG and GO enrichment analysis was performed by clusterProfiler.^65^ The TPM (transcripts per million reads) for genes and transcripts were calculated by RSEM (v1.3.1).^66^

#### Principal components analysis (PCA)

The pseudo-bulk samples were generated by aggregating single-cell counts per gene from each sample. Then we merged and normalized all bulk RNA-seq and pseudo-bulk by library size. To eliminate the technical effect, we used the ComBat in SVA^67^ to minimize the effect between bulk and pseudo-bulk datasets. The corrected values were subjected to PCA.

#### Molecular classifications based on ssGSEA enrichment scores and tumor subtype classification

The subtype classification was based on ssGSEA enrichment scores for bulk and single-cell pseudo-bulk TNP samples at the different stages, using a published protocol.^4^ Briefly, we generated a large number (≥ 100, 000) of random ssGSEA scores for each subtype among the log-transformed expression to build the null distribution and to calculate the empirical *P* values. The subtype with the highest –log_10_ *P* value was considered the sample subtype. The TCGA tumor subtype classification was calculated by GEPIA2 (http://gepia2.cancer-pku.cn).^29^

#### GBM-normal expression comparison analyses

The TPM of the given genes from TCGA GBM and matched GETx normal tissue RNA-seq datasets were visualized by GEPIA2 (http://gepia2.cancer-pku.cn).

#### Primary sphere lineage analysis

Bulk expression profiles of normal lineage cells including astrocytes, neurons, and oligodendrocytes were downloaded from GSE9566.^68^ The Pearson correlation coefficients were calculated between normal lineage cells and TN primary spheres.

#### Deconvolution of TN bulk RNA-seq

We used MuSiC^69^ to deconvolute the transcriptome of TN Bulk RNA-Seq samples into the likely constituent cell types, using TNP scRNA-seq datasets as a reference. We calculated the predicted proportions of each cell type in TN bulk samples, and visualized these proportions with ComplexHeatmap (v2.0.0).^70^

### ScRNA-seq and analysis

#### Library preparation and sequencing

Single cells were prepared in the Chromium Single Cell Gene Expression Solution using the Chromium Single Cell 3′ Gel Bead, Chip and Library Kits v2 (10× Genomics) as per the manufacturer’s protocol. 8000–10,000 total cells were added to each channel with an average recovery of 5758 cells. The cells were then partitioned into Gel Beads in Emulsion in the Chromium instrument, where cell lysis and barcoded reverse transcription of RNA occurred, followed by amplification, shearing 5′ adapter, and sample index attachment. Libraries were sequenced on the Illumina NovaSeq 6000 platform at Novogene, Beijing, China. On average each sample generated about 709 M reads (SD = 121 M).

#### Alignment and quantification

The sequencing data were processed using CellRanger software (version 3.0.0) with default parameters, mapping to the human (hg19), mouse (mm10) genomes, and exogenous genes (e.g., PuroR) introduced by the viral plasmid. Human cells were sorted out based on their alignment to the human genome. Gene expression was quantified based on the unique molecular identifier (UMI) (PuroR > 0). We removed outlier cells by isOuterlier from Scater package (v1.12.2)^71^ or low-quality cells (gene count < 500 or the mitochondria gene ratio >25%).

#### Clustering and annotation

We used Seurat (v3.1.0) for downstream analyses including data normalization (NormalizeData, LogNormalize method, scaling factor 10,000), data feature scaling (ScaleData), variable gene detection (FindVariableGenes with vst method) and PCA of variable genes (RunPCA). The statistically significant PCs were used for Harmony to remove the batch effect, and the two-dimension UMAP was calculated among the Harmony matrix.^30^ Then the original Louvain algorithm (FindClusters) with clustering resolution 0.7 was performed to cluster the cells. We computed DEGs using the FindAllMarkers function in the Seurat package with default parameters.

#### Lineage trajectory analysis

We used a cell lineage inference tool, URD (v1.0.2),^35^ to predict tumor lineage trajectory. We first detected highly variable genes among each stage respectively. To determine the transition probabilities, we calculated the diffusion map with the specific parameter (knn = 200, sigma.use = ’local’)^72^ and defined T0 cells in the NSC1 or NSC2 clusters as the root cells to infer the pseudotime. The URD used a novel diffusion approach that simulates random walks from each tip to the root, and measures the frequency of cells visited by walks from each tip, and the best route was then determined. The tip cells were defined by neurons, astrocytes, and oligodendrocytes at different stages. We computed the lineage-specific genes using the aucprTestAlongTree function in URD with default parameters.

#### Pseudotime in sub-clusters

We first detected highly variable genes for each cluster to calculate the diffusion map with the default parameters, then inferred the pseudotime by defining the cells at T0 as the root cells. The differential expression tests along the pseudotime were performed by differentialGeneTest in monocle (v2.12.0),^73^ and the significantly altered genes (p-adj < 0.01) were selected for downstream analyses. To find genes specifically upregulated at each stage, we calculated the mean values of each gene at different stages, and then classified each gene to a stage by its maximum expression, we further required stage-specific genes with a 1.2-fold-change over other stages.

#### Stemness signature and meta-module score calculation

The genes in the list of stemness signature were obtained from previous publications.^5,8,41^ To calculate the stemness score, we aggregated the average normalized expression of all genes in every module and used this value as the module signatures. The scaled scores were visualized by ComplexHeatmap (v2.0.0).

#### Correlation to NSC-like cells in human GBM datasets

We first isolated the NSC-like cell population from pediatric and adult GBM datasets, and then calculated the Pearson correlation coefficients between NSC-like cells in human GBM and our TNP samples based on stage-specific genes of NSC1 population. The mean values of the correlation coefficients were visualized by ComplexHeatmap (v2.0.0).

#### Stage-specific transcriptional regulatory network in NSC1 population

The stage-specific TFs of the NSC1 population were used to build the gene regulatory network based on the Encode TF-gene interaction database from NetworkAnalyst website.^74^ The JSON format network files were imported to Cytoscape (v3.5.1)^75^ for downstream network analyses. The nodes were sized according to the degree of connectivity and the network was visualized with an organic layout.

#### Human GBM scRNA-seq dataset

We analyzed the scRNA-seq expression profiles of pediatric and adult human GBMs with GEO or Bioproject accession number GSM3828672,^5^ GSE138794,^7^ and PRJNA579593.^6^ For each datasets, we excluded all cells according to the original filter criterion. The annotations of GSM3828672 and PRJNA579593 were downloaded from Broad Single Cell Portal (https://singlecell.broadinstitute.org/single_cell), and UCSC Cell Browser (http://gbm.cells.ucsc.edu), respectively. The malignant cells (GSM3828672 and PRJNA579593) and all cells (GSE138794) were isolated for downstream analyses, including variable gene detection (FindVariableGenes with vst method), PCA among variable genes (RunPCA) and UMAP among PCA matrix (RunUMAP) in Seurat (v.3.1.0).^76^

#### Human hippocampal development scRNA-seq dataset

We analyzed the scRNA-seq expression profiles of the human hippocampus from the GEO with the accession number GSE131258.^38^ Following the original filtering criteria, we kept cells that expressed more than 800 genes and fewer than 7000 genes, and only analyzed genes expressed in at least 30 single cells (0.1% of the total cells) for downstream analyses. The markers *HOPX/PAX6*, *ASCL1*, *NEUROD2*, *GAD1*, *OLIG2*, *MBP*, *AQP4*, *SPARC*, and *PTPRC* were used to mark hippocampal hNSCs, progenitor cells, excitatory neurons, inhibitory neurons, OPCs, oligodendrocytes, astrocytes, endothelial cells, and microglia, respectively. After assigning subpopulation identity, we isolated *HOPX*+ hNSC clusters, calculated the diffusion map using highly variable genes with default parameters, and inferred the pseudotime by defining hNSCs from GW16 as root cells.

### Quantification and statistical analyses

Anatomically comparable sections from control and mutant brains (at least 3 animals for each group) were visualized under ×20 or ×40 magnification using an Olympus BX51 microscope. For each section, at least three images were captured and subjected to quantification using the ImageJ software. Cell numbers and proportions were analyzed by unpaired Student’s *t*-test. For survival analyses, Log-rank test was used to determine the differences between Kaplan–Meier survival curves. *P* < 0.05 is considered statistically significant. All statistical analyses were performed using R (3.6.0) or GraphPad Prism 5 software.

## Supplementary information

Supplementary information, Fig. S1

Supplementary information, Fig. S2

Supplementary information, Fig. S3

Supplementary information, Fig. S4

Supplementary information, Fig. S5

Supplementary information, Fig. S6

Supplementary information, Fig. S7

Supplementary information, Fig. S8

Supplementary information, Fig. S9

Supplementary information, Tables 1-6

## Data Availability

All the raw data of WES and bulk RNA-seq have been deposited in the NCBI BioProject under accession number: PRJNA597654. All raw counts, TPM matrix, the Seurat, and URD object including expression matrix and cell annotation information of single-cell RNA-seq are available in Figshare (10.6084/m9.figshare.11610870).
