# Supplementary material for: Sequential fate-switches in stem-like cells drive the tumorigenic trajectory from human neural stem cells to malignant glioma
Source: Cell Res. 2021 Jan 4;31(6):684–702. doi: 10.1038/s41422-020-00451-z (PMC8169837; doi:10.1038/s41422-020-00451-z)
Supplement: Supplementary file 1 — Supplementary information, Fig. S1 [file 41422_2020_451_MOESM1_ESM.pdf]

Supplementary information, Fig. S1

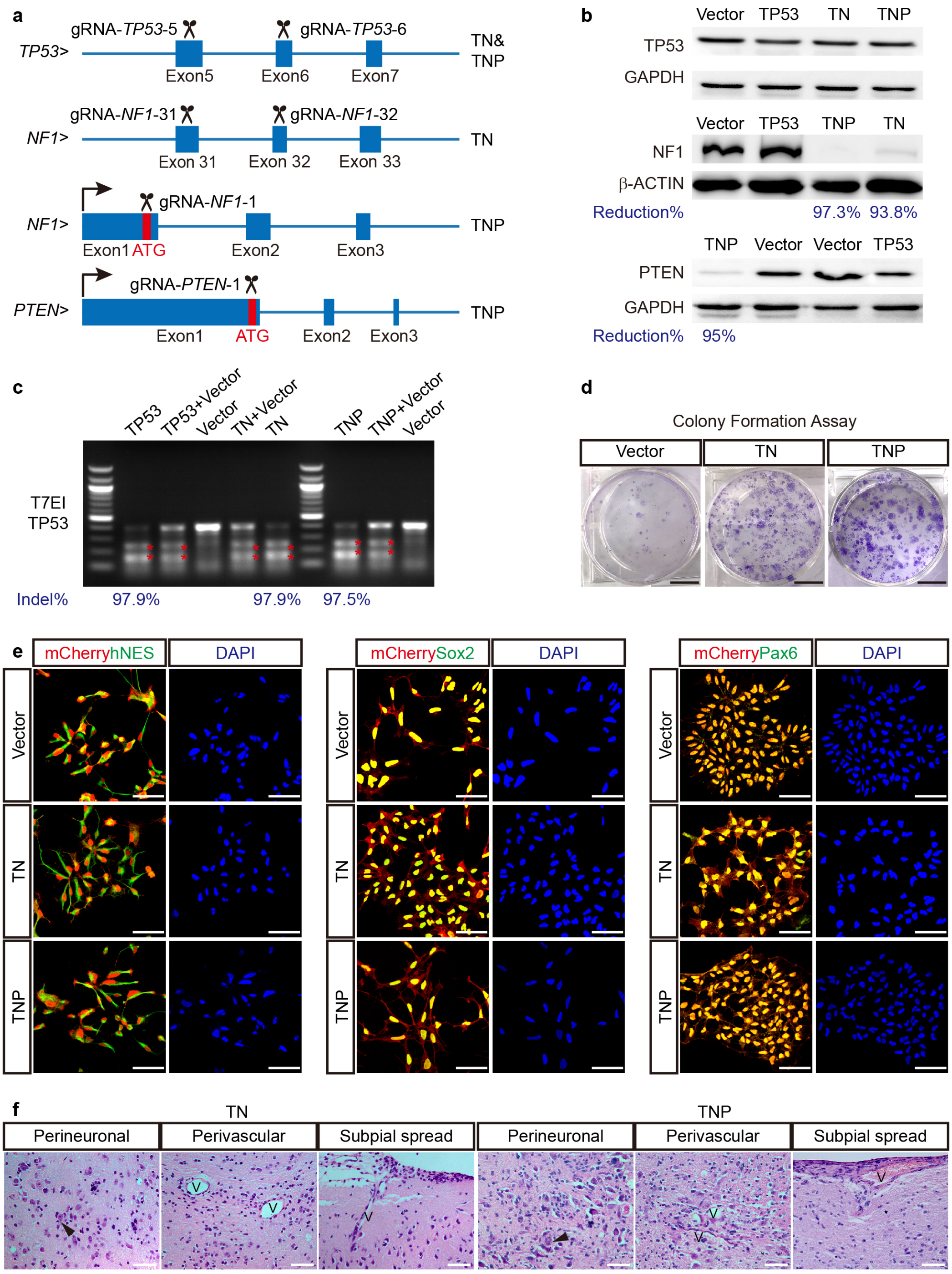

Supplementary information, Fig. S1. The establishment and validation of Vector, TN, and TNP hNSCs.

(**a**) Genome-editing strategy for *TP53, NF1*, and *PTEN* in TN and TNP *g*roups. Scissors, gRNA-target sites. Red rectangles, translation initiation sites. Blue rectangles, exons. TN gRNA combination targets exons 5 and 6 of *TP53* encoding an essential region of the TP53 DNA-binding domain, along with exons 31 and 32 of *NF1* which were frequently mutated in patients and critical for NF1 protein expression. TNP gRNA combination targets the same exonic sites of *TP53,* along with translation initiation sites of *NF1* and *PTEN,* mimicking complete loss of function.

(**b**) Western blot analysis for *TP53*, *NF1*, *PTEN* expression in Vector, *TP53*-single mutant (TP53), TN, and TNP hNSCs. β-ACTIN or GAPDH were used as loading controls. The signal intensity for each band was measured in ImageJ, and the reduction ratios in TN and TNP hNSCs compared to Vector NSCs were calculated.

(**c**) T7EI assay in Vector, TP53, TN, and TNP hNSCs. Samples containing 50% Vector hNSCs PCR products are included as controls to demonstrate the dosage effect. Red asterisks, the cleaved bands. Indel ratios are indicated at the bottom.

(**d**) Colony formation assay for Vector, TN, and TNP hNSCs.

(**e**) IF co-labeling of hNES, Pax6, and Sox2 with mCherry in cultured Vector, TN, and TNP hNSCs.

(**f**) H&E staining for tumor cells invading surrounding tissues in TN and TNP brains at the end stage, highlighting their secondary structures of Scherer (perineuronal satellitosis, perivascular satellitosis, and subpial spread). Arrows, the neuronal nuclei. V, blood vessels.

Scale bars: (**d**) 1cm; (**e, f**) 50μm.
