# Supplementary material for: Sequential fate-switches in stem-like cells drive the tumorigenic trajectory from human neural stem cells to malignant glioma
Source: Cell Res. 2021 Jan 4;31(6):684–702. doi: 10.1038/s41422-020-00451-z (PMC8169837; doi:10.1038/s41422-020-00451-z)
Supplement: Supplementary file 2 — Supplementary information, Fig. S2 [file 41422_2020_451_MOESM2_ESM.pdf]

Supplementary information, Fig. S2

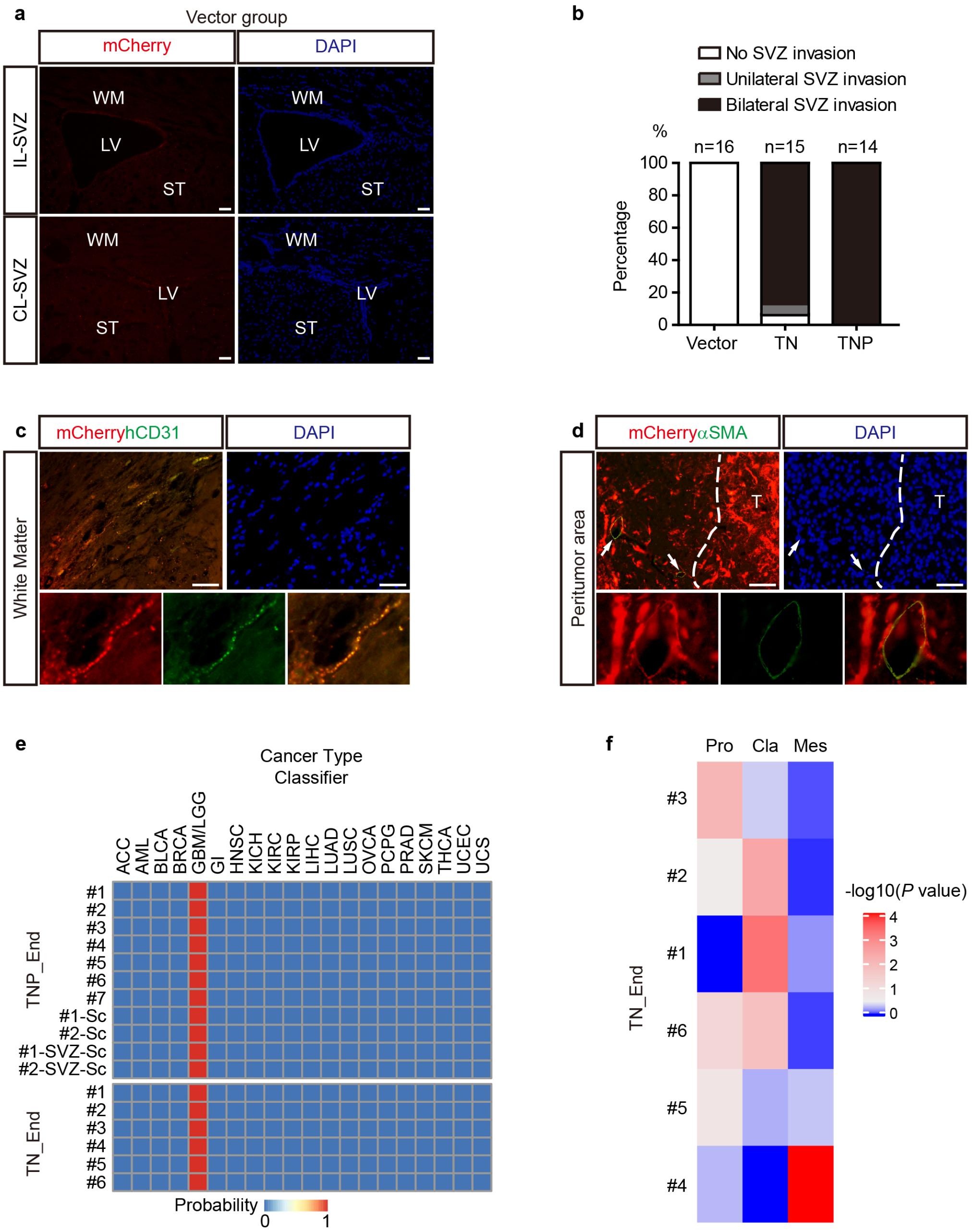

Supplementary information, Fig. S2. TN and TNP tumors exhibit typical features of human GBM.

(**a**) IF for mCherry in the SVZ ipsilateral (IL) and contralateral (CL) to the transplantation sites in brain sections of end-stage Vector mice. WM, white matter. LV, lateral ventricle. ST, striatum.

(**b**) The percentages of Vector, TN, and TNP mice at the end stage exhibiting unilateral or bilateral SVZ invasion.

(**c, d**) IF for mCherry/hCD31(**c**) and mCherry/α-SMA(**d**) in the tumor-infiltrated white matter or peritumor regions in the brains of TN and TNP mice. Arrows, co-labeled cells. T, tumor. Dashed lines, the boundary between the tumor core area and the peritumor striatum region.

(**e**) Heatmap of the cancer subtype probability for end-stage TN and TNP samples based on GEIPA2. Rows, individual samples. Columns, TCGA tumors.

(**f**) Heatmap of the *P* values ($-\log_{10}$) of GBM subtypes for bulk TN samples at End stage based on ssGSEA analysis.

Scale bars: 50μm.
