# Supplementary material for: Sequential fate-switches in stem-like cells drive the tumorigenic trajectory from human neural stem cells to malignant glioma
Source: Cell Res. 2021 Jan 4;31(6):684–702. doi: 10.1038/s41422-020-00451-z (PMC8169837; doi:10.1038/s41422-020-00451-z)
Supplement: Supplementary file 3 — Supplementary information, Fig. S3 [file 41422_2020_451_MOESM3_ESM.pdf]

Supplementary information, Fig. S3

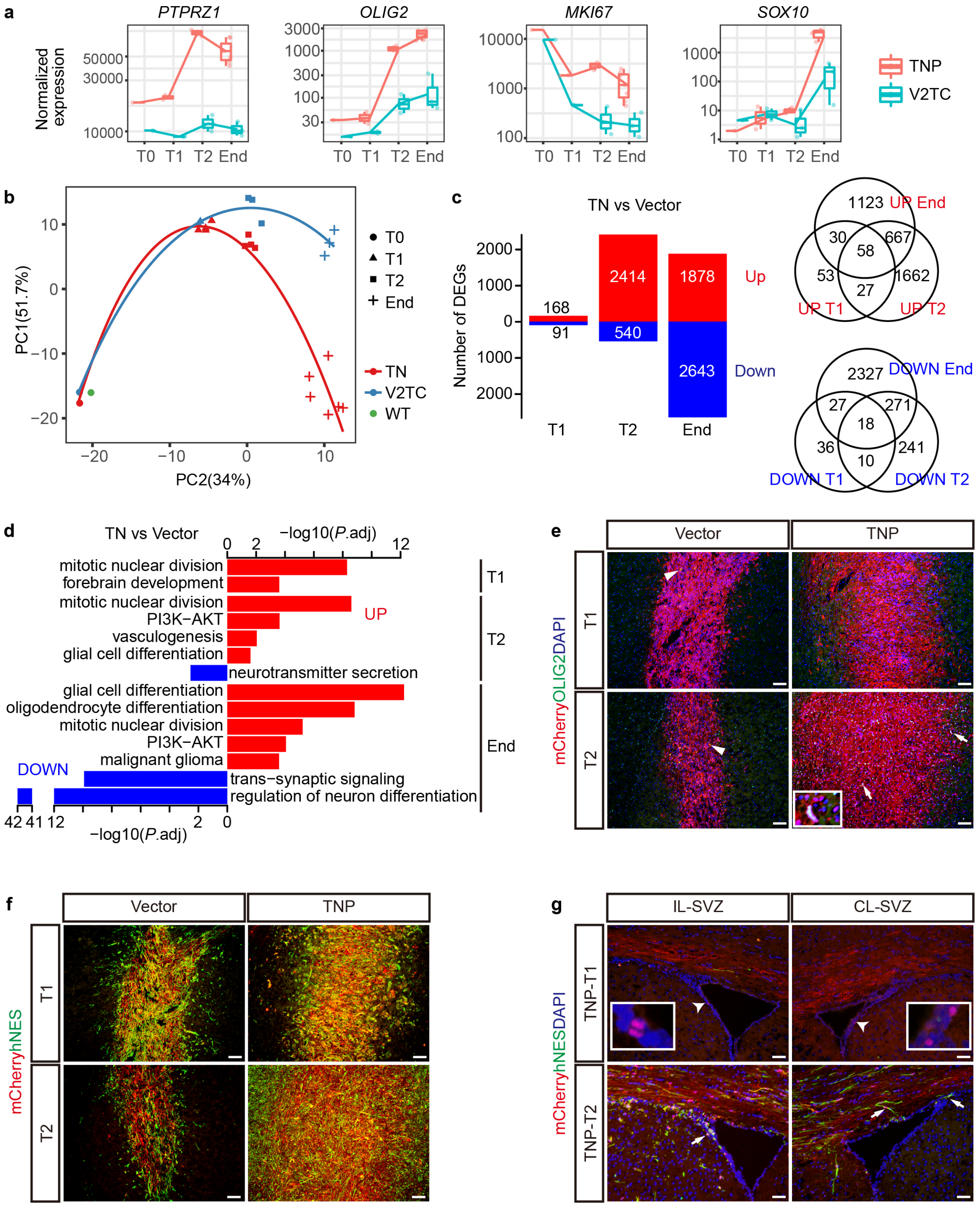

Supplementary information, Fig. S3. Transcriptomic dynamics of TN and TNP tumors at different stages.

(**a**) The normalized temporal expression of representative genes for each stage in Vector and TNP samples.

(**b**) PCA of bulk RNA-seq of WT, Vector, and TN samples at different stages. Note that we rotated the PC1 and PC2 to be consistent with Fig.3b. Dots and curves are colored by sample groups. Shapes represent different stages.

(**c**) Left: The number of DEGs in TN versus Vector at each stage. Right: Venn diagrams illustrating the relationship of significantly upregulated (top) or downregulated (bottom) genes in TN versus Vector samples at each stage.

(**d**) Gene ontology enrichment analyses of DEGs in TN versus Vector at each stage showing representative terms and adjusted *P* values ($-\log_{10}$).

(**e, f**) IF for mCherry/Olig2/DAPI (**e**) and mCherry/hNES (**f**) in the central areas of Vector and TNP cells at T1 and T2. Inset, $mCherry^+/Olig2^+$ cells lining the blood vessel with abnormal nuclei. Triangular arrowheads, autofluorescent debris without DAPI staining.

(**g**) IF for mCherry/hNES/DAPI in the SVZ ipsilateral (IL-SVZ) and contralateral (CL-SVZ) to the transplantation sites of TNP brains at T1 and T2. Arrowheads and inset, rare $mCherry^+/hNES^-$ TNP cells in the SVZ at T1. Arrows, co-labeled cells. Scale bars: 100μm.
