# Supplementary material for: Sequential fate-switches in stem-like cells drive the tumorigenic trajectory from human neural stem cells to malignant glioma
Source: Cell Res. 2021 Jan 4;31(6):684–702. doi: 10.1038/s41422-020-00451-z (PMC8169837; doi:10.1038/s41422-020-00451-z)
Supplement: Supplementary file 4 — Supplementary information, Fig. S4 [file 41422_2020_451_MOESM4_ESM.pdf]

Supplementary information, Fig. S4

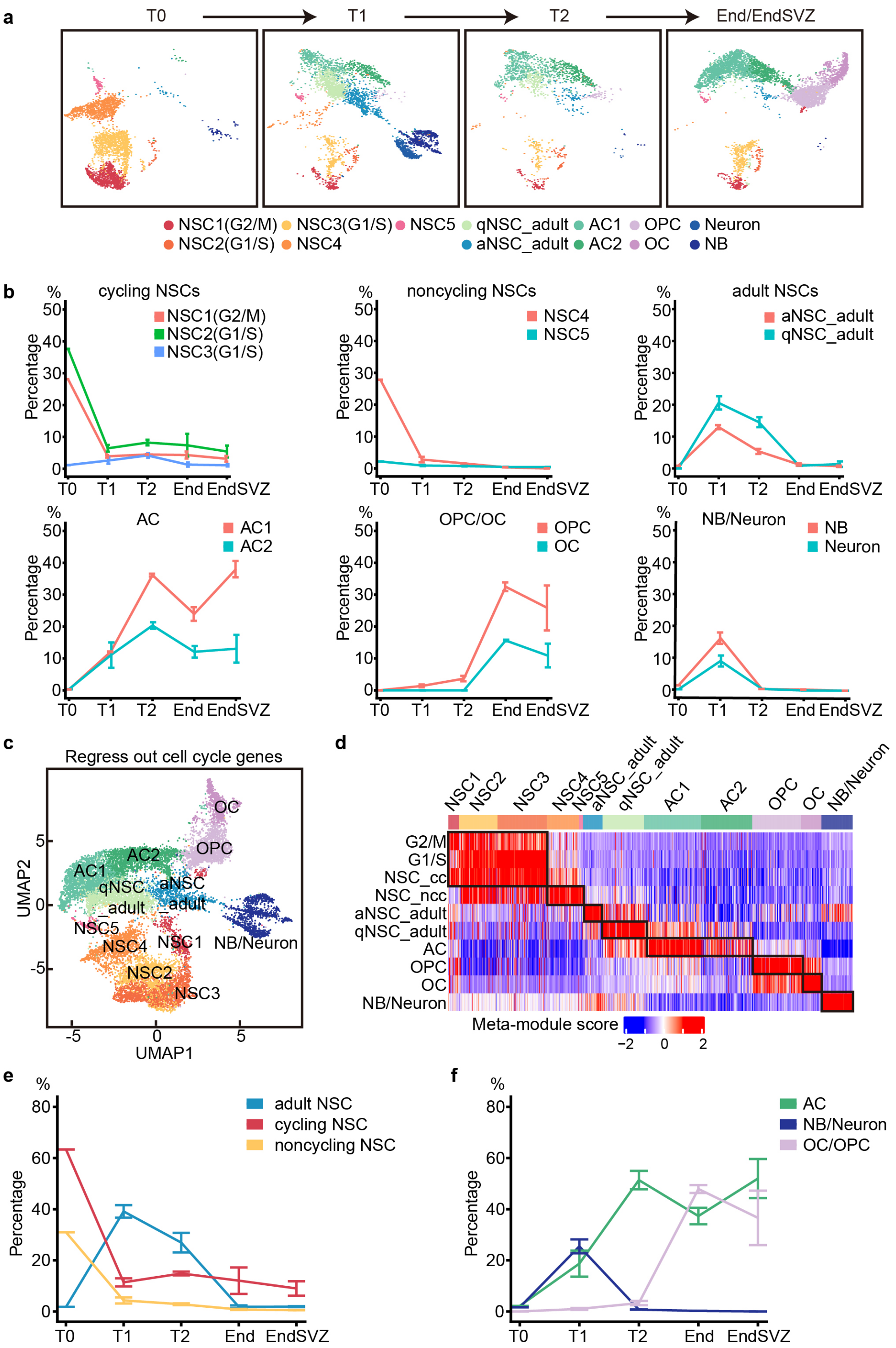

Supplementary information, Fig. S4. The temporal dynamics of TNP subpopulations is not significantly influenced by cell cycle variance.

(**a**) The chronological UMAP plots (from left to right) for TNP single cells at T0 to End/EndSVZ. Clusters are separated by colors.

(**b**) The cellular frequencies of every cluster in Fig. 4a at different stages. Error bars, mean ± SEM.

(**c**) UMAP of human single cells from TNP samples after regressing out the cell cycle genes. Clusters are separated by colors.

(**d**) Heatmap of scaled meta-module scores for cells in each cluster in (**c**).

(**e**) The cellular frequencies of NSC subpopulations per sample at different stages after regressing out cell cycle genes. Error bars, mean ± SEM.

(**f**) The cellular frequencies of AC, OPC/OC, and NB/Neuron subpopulations per sample at different stages after regressing out cell cycle genes. Error bars, mean ± SEM.
