# Supplementary material for: Sequential fate-switches in stem-like cells drive the tumorigenic trajectory from human neural stem cells to malignant glioma
Source: Cell Res. 2021 Jan 4;31(6):684–702. doi: 10.1038/s41422-020-00451-z (PMC8169837; doi:10.1038/s41422-020-00451-z)
Supplement: Supplementary file 5 — Supplementary information, Fig. S5 [file 41422_2020_451_MOESM5_ESM.pdf]

Supplementary information, Fig. S5

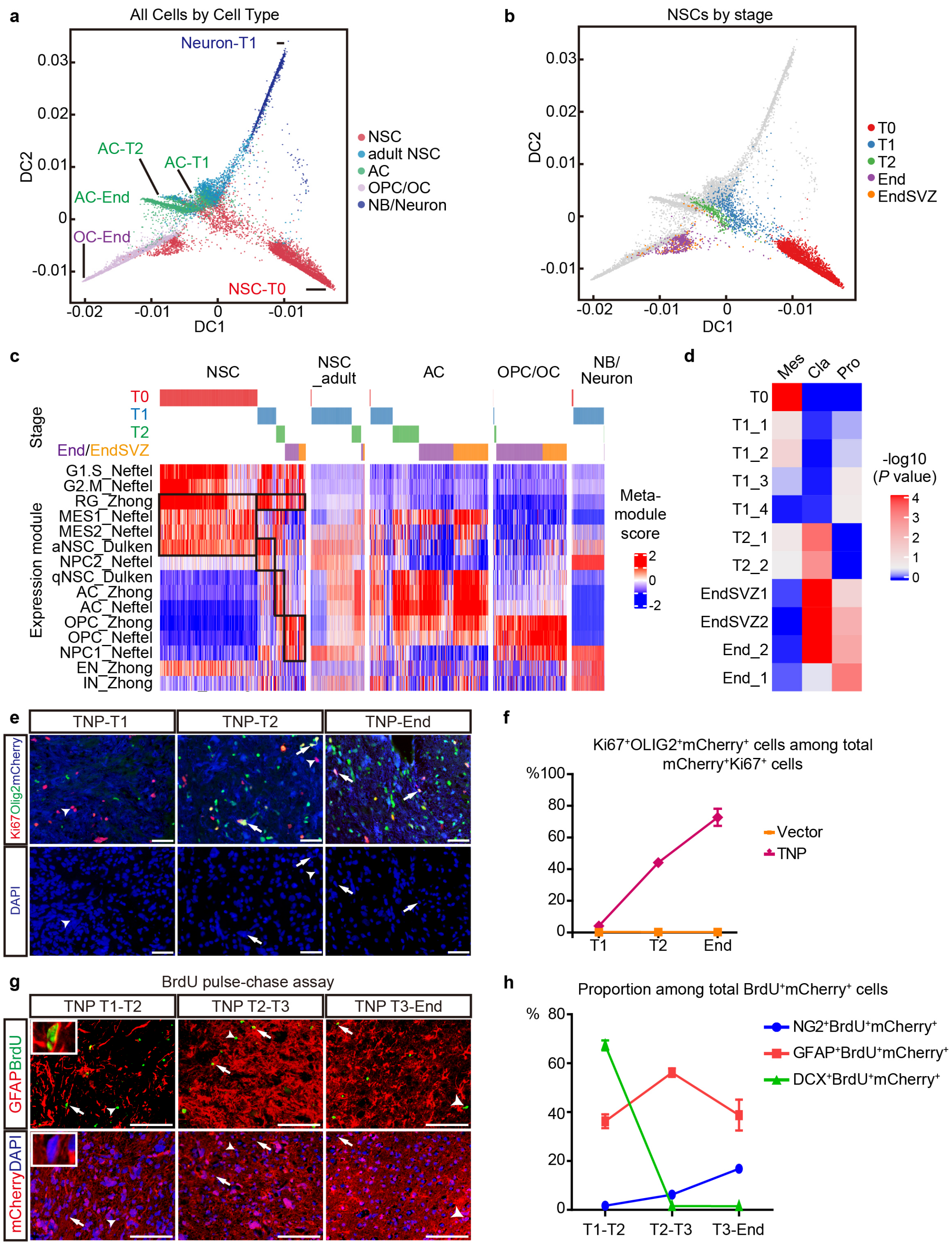

Supplementary information, Fig. S5. Diffusion map of TNP single cells.

(**a**) Diffusion map of TNP single cells. Cells are colored by cell type with tips for each branch labeled.

(**b**) NSCs at different stages highlighted in the diffusion map.

(**c**) Heatmap of scaled meta-module scores for cells in each subpopulation ordered by stage.

(**d**) Heatmap of the *P* values ($-\log_{10}$) of GBM subtypes for single-cell pseudo-bulk of TNP samples at the T1, T2, and End stages based on ssGSEA analysis.

(**e**) Triple-labeling of Ki67/Olig2/mCherry in the central areas of TNP cells at T1, T2 and End. Arrows, triple-positive cells. Arrowheads, $Ki67^+Olig2^-mCherry^+$ cells. Scale bars: 50μm.

(**f**) The ratio of $Ki67^+Olig2^+mCherry^+$ cells among total $Ki67^+mCherry^+$ cells in Vector and TNP brains at T1, T2, and End.

(**g**) IF co-labeling of mCherry with GFAP/BrdU in the central regions of TNP cells o pulsed at T1, T2, and T3 and analyzed at T2, T3, and End, respectively. Arrows, triple-positive cells. Arrowheads, $BrdU^+GFAP^-mCherry^+$ cells. Inset, a representative $BrdU^+GFAP^+mCherry^+$ cell. Scale bars: 100μm.

(**h**) The proportion of $NG2^+BrdU^+mCherry^+$, $GFAP^+BrdU^+mCherry^+$, or $DCX^+BrdU^+mCherry^+$ cells among total $BrdU^+mCherry^+$ cells in BrdU pulse-chase assays.
