# Supplementary material for: Sequential fate-switches in stem-like cells drive the tumorigenic trajectory from human neural stem cells to malignant glioma
Source: Cell Res. 2021 Jan 4;31(6):684–702. doi: 10.1038/s41422-020-00451-z (PMC8169837; doi:10.1038/s41422-020-00451-z)
Supplement: Supplementary file 6 — Supplementary information, Fig. S6 [file 41422_2020_451_MOESM6_ESM.pdf]

Supplementary information, Fig. S6

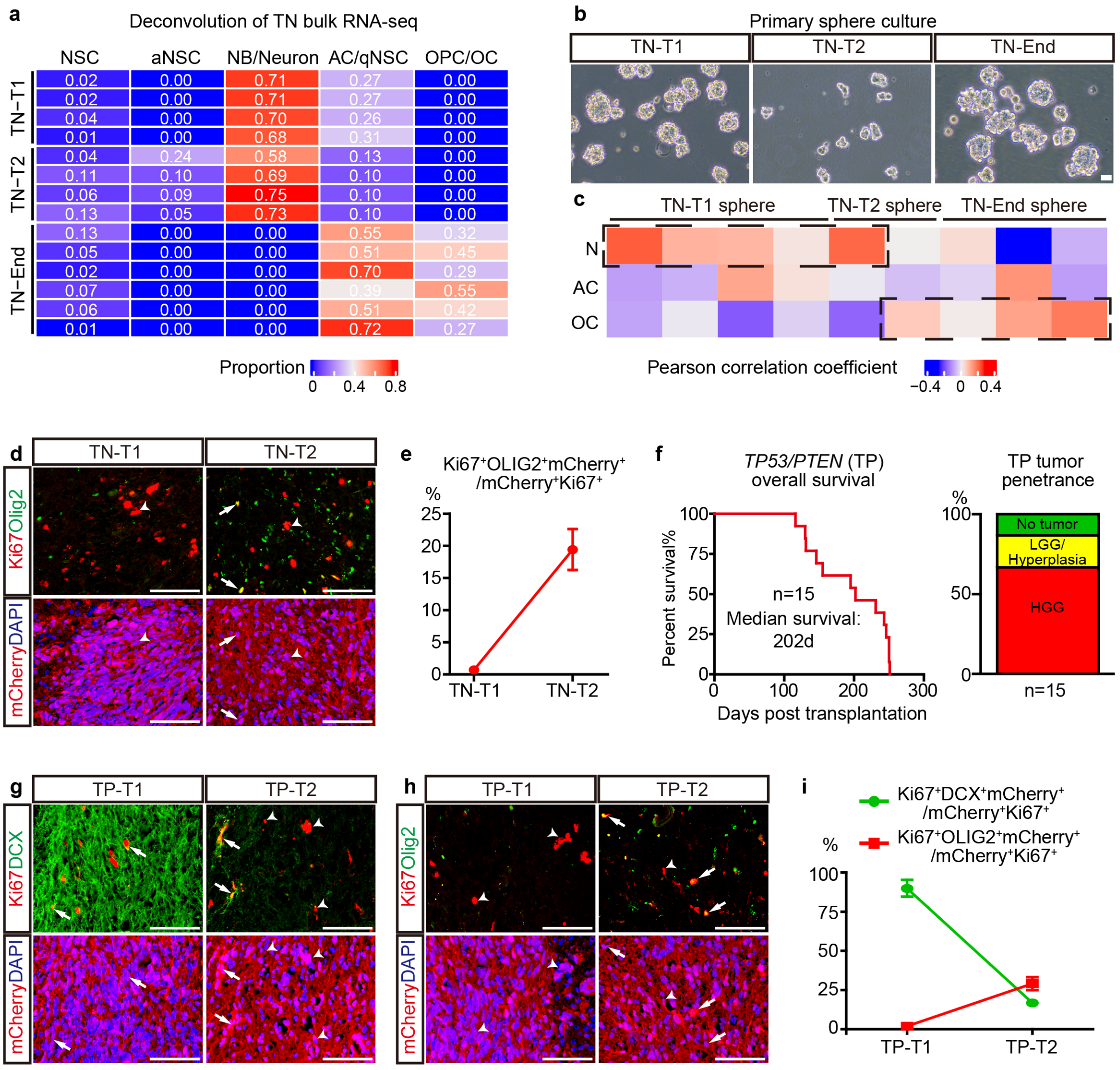

Supplementary information, Fig. S6. Cellular composition alterations and fate-switches during gliomagenesis in TN and TP models.

(**a**) Heatmap of the predicted proportion of each cell type (columns) of TN bulk RNA-seq samples (rows) using deconvolution methods based on TNP reference cell types.

(**b**) Dissociated $mCherry^+$ tissue samples from TNP mice at T1, T2, and End form spheres under the non-adherent stem cell culture condition. Images representative of 2-4 biological replicates at each stage.

(**c**) Heatmap of the Pearson correlation coefficients between normal lineage cells (rows) and cultured TN spheres at different stages (columns).

(**d**) IF co-labeling of mCherry with Ki67/Olig2 in the central regions of TN cells at T1 and T2. Arrows, $Ki67^+Olig2^+mCherry^+$ cells. Arrowheads, $Ki67^+Olig2^-mCherry^+$ cells. Scale bars: 50μm.

(**e**) The ratio of $Ki67^+Olig2^+mCherry^+$ cells among total $Ki67^+mCherry^+$ cells in TN brains at T1 and T2.

(**f**) Left: the overall survival curve of TP mice. n, the number of animals. Right: the percentage of TP mice diagnosed with no tumor, LGG/hyperplasia, and HGG.

(**g**, **h**) IF co-labeling of mCherry with Ki67/DCX (**g**) and Ki67/Olig2 (**h**) in the central regions of TP cells at T1 and T2. Arrows, double-positive cells. Arrowheads, $Ki67^+DCX^-mCherry^+$ cells (**g**) or $Ki67^+Olig2^-mCherry^+$ (**h**) cells. Scale bars: 50μm.

(**i**) The ratios of $Ki67^+DCX^+mCherry^+$ or $Ki67^+Olig2^+mCherry^+$ cells among total $Ki67^+mCherry^+$ cells in TP mice brains at T1 and T2. Of note, at T2 there were TP samples that did not exhibit cellular expansion and were comparable to vector controls. These samples were excluded from this analysis.
