# Supplementary material for: Sequential fate-switches in stem-like cells drive the tumorigenic trajectory from human neural stem cells to malignant glioma
Source: Cell Res. 2021 Jan 4;31(6):684–702. doi: 10.1038/s41422-020-00451-z (PMC8169837; doi:10.1038/s41422-020-00451-z)
Supplement: Supplementary file 7 — Supplementary information, Fig. S7 [file 41422_2020_451_MOESM7_ESM.pdf]

Supplementary information, Fig. S7

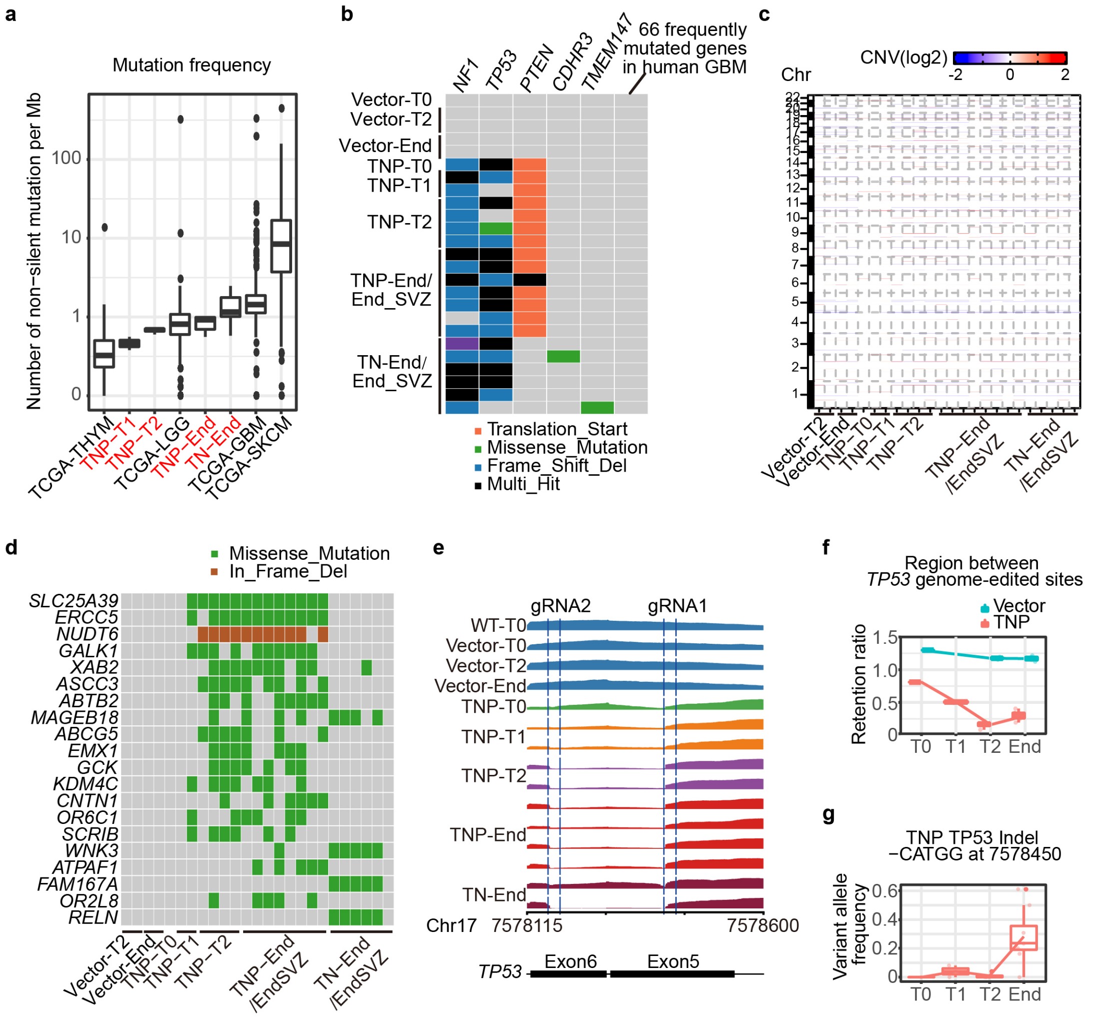

Supplementary information, Fig. S7. Genomic alterations of TN and TNP samples at each stage.

(**a**) The frequency of non-silent mutations in TN and TNP samples at T1, T2, and End compared to selected TCGA tumor types THYM, LGG, GBM, and SKCM.

(**b**) The mutational status of 71 frequently mutated genes in human GBM including *TP53*, *NF1*, and *PTEN* among Vector, TN, and TNP samples at different stages. Columns, samples; rows, genes.

(**c**) Inferred CNVs in Vector, TN, and TNP samples at different stages based on WES data. Columns, individual samples. Rows, chromosomes.

(**d**) The mutational status of recurrently mutated genes in Vector, TN, and TNP samples at different stages.

(**e**) WES read densities of genomic regions covering *TP53* exons 5 and 6 in WT, Vector, TN, and TNP samples at different stages. Dashed lines, regions targeted by TP53 gRNAs.

(**f**) The retention ratio of the genomic region between two gene-edited sites in Vector and TNP samples at different stages.

(**g**) The variant allele frequency for a TP53 mutant in TNP samples with a short deletion of CATGG in TP53 exon 5.
