# Supplementary material for: Sequential fate-switches in stem-like cells drive the tumorigenic trajectory from human neural stem cells to malignant glioma
Source: Cell Res. 2021 Jan 4;31(6):684–702. doi: 10.1038/s41422-020-00451-z (PMC8169837; doi:10.1038/s41422-020-00451-z)
Supplement: Supplementary file 8 — Supplementary information, Fig. S8 [file 41422_2020_451_MOESM8_ESM.pdf]

Supplementary information, Fig. S8

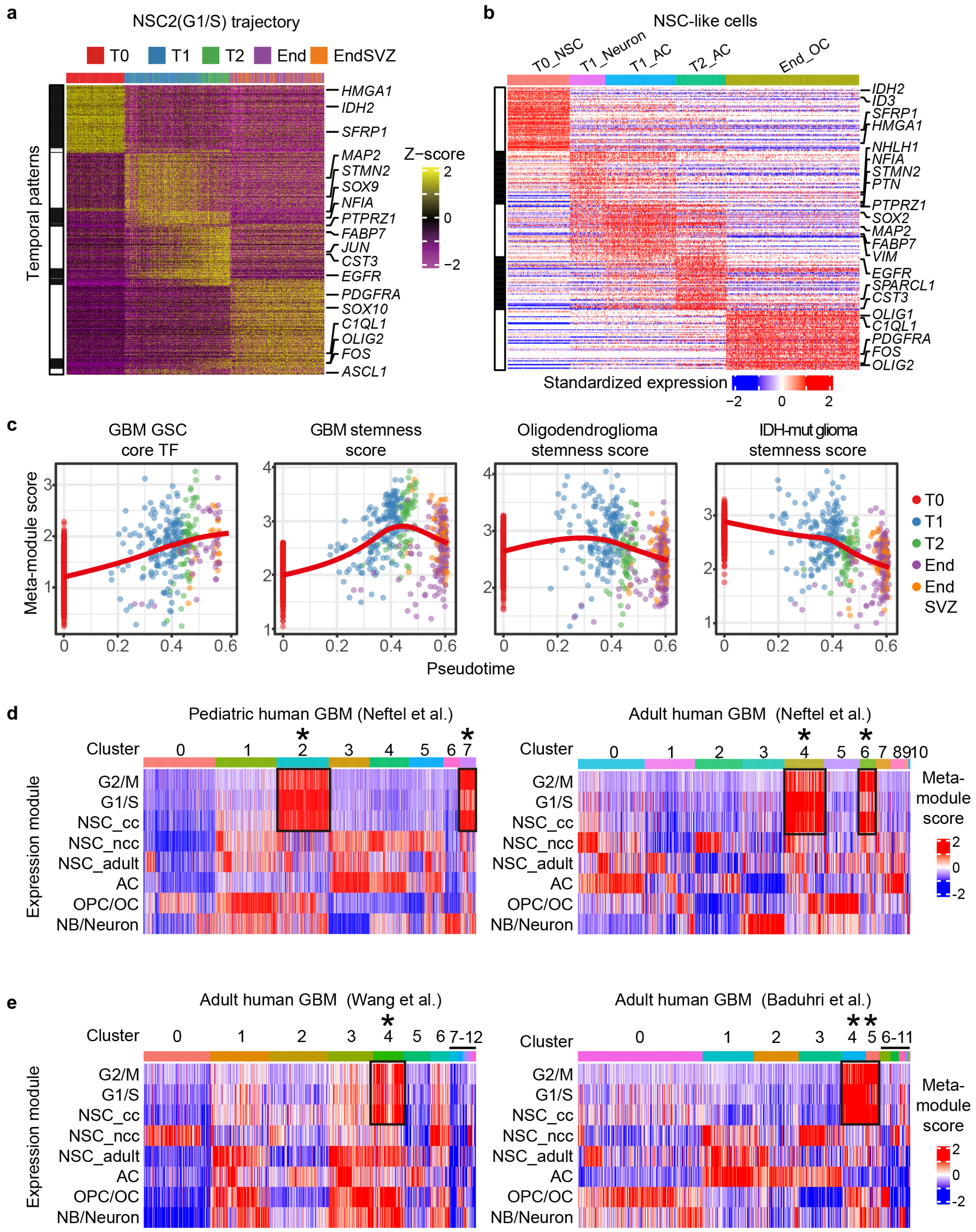

Supplementary information, Fig. S8. Cell-type- and stage-specific signatures of NSC-like cells and their presence in human pediatric and adult GBM samples.

(**a**) Heatmap of gene expression cascades along the pseudotime of NSC2. Columns, pseudotime-ordered cells. Rows, genes. Colors in the top row indicate the actual sample time of each cell. Genes were clustered by their temporal expression patterns (left bars).

(**b**) The heatmap of DE genes in NSC-like cells located in different branches of the gliomagenesis tree (Figure 5**a, c**).

(**c**) Trajectory plots of the meta-module score of published glioma stemness gene sets along the pseudotime of NSC1.

(**d, e)** Heatmap of the meta-module scores for single tumor cells of pediatric and adult GBMs from Neftel et al. (**d**), Wang et al. and Baduhri et al. (**e**) studies. The cells clusters were defined by unsupervised clustering in UMAP. Asterisks, NSC-like subpopulations.
