# Supplementary material for: Sequential fate-switches in stem-like cells drive the tumorigenic trajectory from human neural stem cells to malignant glioma
Source: Cell Res. 2021 Jan 4;31(6):684–702. doi: 10.1038/s41422-020-00451-z (PMC8169837; doi:10.1038/s41422-020-00451-z)
Supplement: Supplementary file 9 — Supplementary information, Fig. S9 [file 41422_2020_451_MOESM9_ESM.pdf]

Supplementary information, Fig. S9

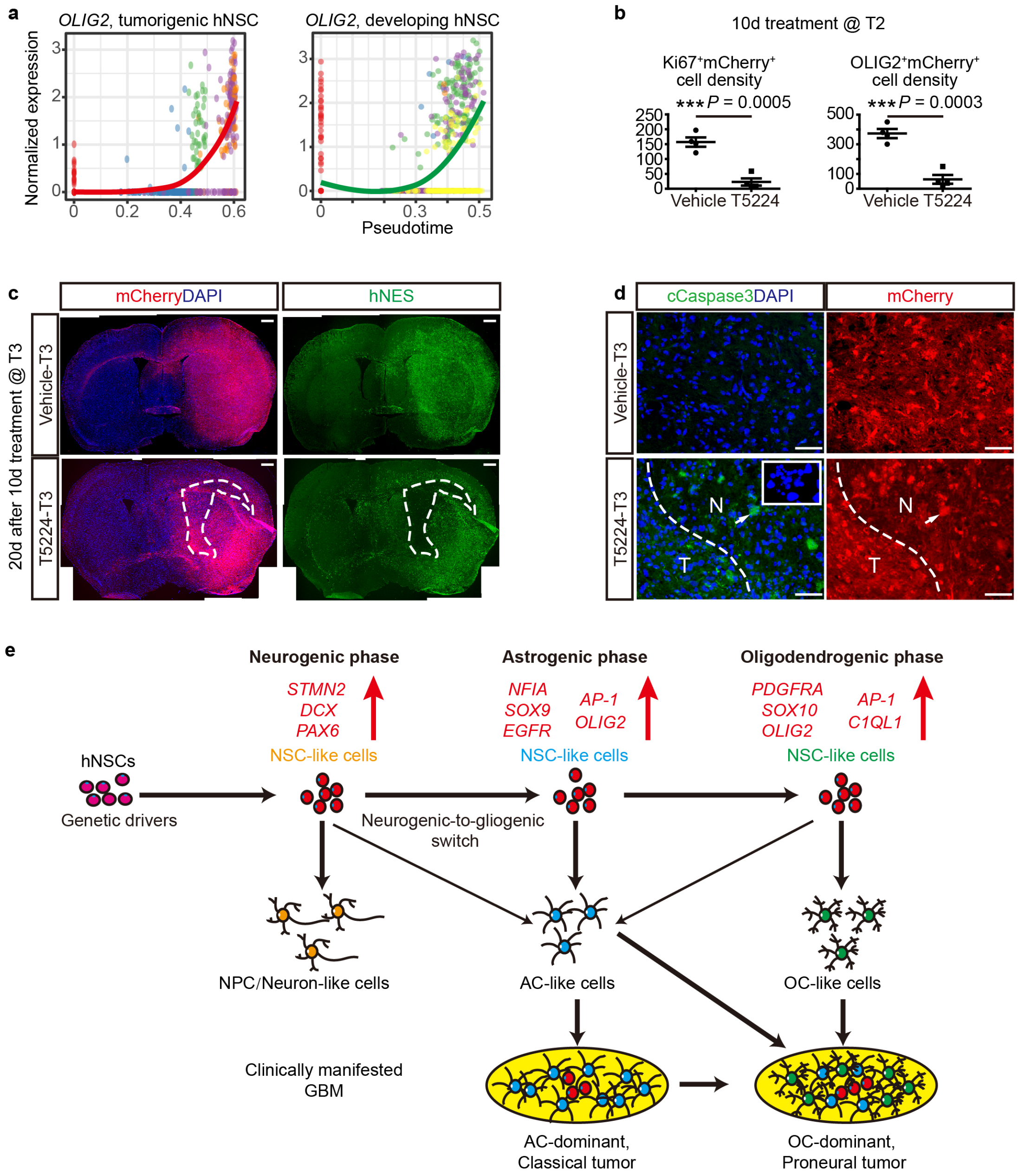

Supplementary information, Fig. S9. AP-1 inhibition at a later stage exhibited partial tumor inhibition.

(**a**) Trajectory plots of *OLIG2* in tumorigenic TNP hNSCs (top) and developing embryonic hNSCs (bottom).

(**b**) The number of $Ki67^+mCherry^+$, and $OLIG2^+mCherry^+$ cells per high-power field in the central tumor areas of TNP mice treated with vehicle or T5224 at T2 for 10 days. n=4 for each group, *P* values from Student's t-test.

(**c**) Low magnification view of $mCherry^+DAPI^+$ cells and $hNES^+$ cells in brain sections of TNP mice 20 days after the 10-day treatment with vehicle or T5224 at T3. Dashed lines, the overt necrotic region inside the tumor. Scale bars, 1mm.

(**d**) IF for cCaspas3/DAPI and mCherry in brain sections of TNP mice 20 days after the 10-day treatment with vehicle or T5224 at T3. Dashed lines, a rough boundary between the necrotic region and the remaining tumor region. Inset, apoptotic nucleus. N, necrotic region. T, tumor region. Scale bars, 50μm.

(**e**) A proposed model summarizing the tumorigenic trajectory from mutant hNSCs to clinically manifested GBM. Genes in red represent signature genes in NSC-like cells at each phase.
